# Cross‐Species Plasticity and Divergence Under Alkaline Soil Signature Conditions Differentiating PYE From FIT Target Transcripts in the *Arabidopsis thaliana* Ferrome

**DOI:** 10.1111/pce.70645

**Published:** 2026-06-04

**Authors:** Gen Yang, Hans Simon Metze, Ute Krämer

**Affiliations:** ^1^ Molecular Genetics and Physiology of Plants, Faculty of Biology and Biotechnology Ruhr University Bochum Bochum Germany

**Keywords:** acidification, alkalinization, Fe acquisition, Fe‐deficiency induced transcription factor, H^+^‐ATPase 7 (AHA7), IAA‐leucine resistant 3 (ILR3), iron nutrition, POPEYE, rhizosphere, root morphology

## Abstract

Soil pH is an influential abiotic environmental factor. Here, we identify responses to central stimuli associated with calcareous alkaline soils in *Arabidopsis* and comparatively explore the potential of the closely related *Arabidopsis halleri* for addressing how plants cope with local soil pH. We profiled the root transcriptome upon applying a set of hydroponic treatments—low bioavailable iron (Fe), alkaline pH and both in combination. Among the known Fe deficiency responses (ferrome), pervasively Fe‐deficiency responsive transcripts (pIDR) encoding the transcription factor PYE and its targets responded co‐directionally to Fe deficiency also at pH 7.5. A distinct subgroup of additionally alkaline‐pH‐responsive transcripts (aAPRs), comprising FIT and FIT‐dependent genes, showed a co‐directional response to pH 7.5 even with available Fe. Root hair densities in both species increased at an alkaline pH. Compared with *A. thaliana*, *A. halleri* accessions from soils of contrasting pH showed elevated tolerance to low‐Fe and alkaline pH based on chlorophyll content and root growth. *A. halleri* from high‐pH soils convergently exhibited longer and less branched root systems, more stable ionomes across conditions, and attenuated root growth at alkaline pH with Fe. Our findings provide insights into Fe‐deficiency regulatory networks and identify soil pH‐related phenotypic divergence within *A. halleri* and across species.

## Summary statement

Plants exposed to stimuli encountered on alkaline soils orchestrate fine‐tuned re‐organization that involves distinct responses of two regulatory sub‐networks of the Arabidopsis ferrome. *Arabidopsis halleri* is a promising model for addressing the molecular bases of parallel and convergent adaptations to local soil pH.

## 1 Introduction

Soil pH is known as a parameter of high predictive value in plant ecology and agriculture (Bontpart et al. 2024; Ellenberg 1958; Marschner 1995; Tsai and Schmidt 2021). Soil pH values between 5.5 and 7 are considered optimal for plant growth. Outside this range, both acidic (low pH < 5.5) and alkaline (high pH > 7) soils constitute challenging edaphic environments, primarily because of their nutritionally imbalanced content in bioavailable ions. Alkaline soils are mostly calcareous, meaning that they contain more than 15% and up to 95% calcium carbonate (CaCO_3_) (Bontpart et al. 2024; Marschner 1995). Calcareous alkaline and acidic soils each comprise 30% or more of the worldwide topsoils, respectively (Marschner 1995; Kochian et al. 2004). Acidic and alkaline soils host natural plant communities of distinctive species composition, and the yields of sensitive agricultural crops can be strongly diminished on such soils (Bontpart et al. 2024; Ellenberg 1958; Marschner 1995; Tsai and Schmidt 2021).

In acidic soils, proton and aluminium (Al) toxicity are the predominant factors affecting plant growth (Kochian et al. 2004). Beyond this, the bioavailability of Mn is increased, that of Mo is decreased, and the levels of the macronutrients N, P, K, Mg and Ca tend to be low. The most common symptom of stress in crop plants cultivated on calcareous soils is ‘lime‐induced chlorosis’ resulting from Fe deficiency (Marschner 1995). Strongly impaired iron (Fe^III^) bioavailability in calcareous soils is thought to result from the combination of a high soil pH and the strong pH‐buffering capacity of bicarbonate (HCO_3_^−^) in the soil solution. With every unit increase in soil pH, the levels of Fe^3+^ in the soil solution decrease by a factor of approximately 1000 (Palmer and Guerinot 2009). Additionally and with less severe consequences, the high soil pH of calcareous soils also interferes with the bioavailability of Mn, Zn, P and B, whereas soil Ca levels are very high (Bontpart et al. 2024; Marschner 1995; Tsai and Schmidt 2021). To date, we know little about the genetic loci, molecular mechanisms, and traits underlying evolutionary adaptations of plants to soil pH, and related work in crops has primarily focused on acidic soils to date (Tsai and Schmidt 2021; Poormohammad Kiani et al. 2012; Maron et al. 2013; Almira Casellas et al. 2023; Pérez‐Martin et al. 2024; Guggisberg et al. 2018; Gujas et al. 2012).

Considering that calcareous soils predominantly affect plant Fe nutrition, we can build on existing knowledge of Fe acquisition as well as plastic responses to Fe deficiency in the model plant *Arabidopsis thaliana* (Gao et al. 2019; Brumbarova et al. 2015; Palmer and Guerinot 2009). Alongside all angiosperm plants except the grasses, Arabidopsis pursues Strategy I of Fe acquisition. Accordingly, plants solubilize Fe^III^ by acidifying the root surface in the root hair zone, the principal site of plant Fe acquisition, with a predominant role of the plasma membrane proton pump Arabidopsis H^+^‐ATPase 2 (AHA2) (Santi and Schmidt 2009). The root surface ferric (Fe^III^) chelate reductase named ferric reduction oxidase 2 (FRO2) acts to shuttle electrons from the cytosol across the plasma membrane for the formation of more highly soluble ferrous (Fe^2+^) cations, which are subsequently taken up into root cells predominantly via the high‐affinity Fe uptake system iron regulated transporter 1 (IRT1) (Robinson et al. 1999; Connolly et al. 2002; Vert et al. 2002). To promote Fe mobilization in the rhizosphere, Arabidopsis roots synthesize and secrete chelator molecules of the coumarin family, which they export from root cells through pleotropic drug resistance 9 (PDR9, ABCG37) and which include catechol coumarins that can react with Fe^3+^ to form Fe^2+^ (Fourcroy et al. 2014; Rodriguez‐Celma et al. 2013; Schmid et al. 2014; Schmidt et al. 2014). All these processes are upregulated in Fe‐deficient plants irrespective of the pH in the rhizosphere, dependent on a hierarchically organized network of transcription factors and regulatory proteins acting to increase transcript levels of the corresponding genes (Spielmann et al. 2023; Gao et al. 2019).

Fewer studies have addressed acclimative responses of Arabidopsis to high pH, a second central characteristic feature of calcareous soils. By comparison to Fe deficiency alone, coumarin secretion by roots is more strongly increased in seedlings cultivated at an alkaline pH with no Fe or with insoluble forms of Fe^III^, and the biosynthesis of catechol coumarins is modulated to enhance the production of fraxetin at the cost of sideretin formation (Paffrath et al. 2024). The formation of Fe^III^‐fraxetin complexes at circumneutral pH facilitates Fe mobilization and can partially alleviate Fe deficiency even in an *IRT1* loss‐of‐function genetic background in Arabidopsis (Robe et al. 2025).

In addition to abundant information on plant transcriptomic responses to Fe deficiency at ordinary/optimal pH (Kim et al. 2019), which are addressed as the ferrome (Mcinturf et al. 2022; Schmidt and Buckhout 2011), some studies examined how plants may cope with calcareous soils by analyzing responses to high pH or bicarbonate (Pérez‐Martin et al. 2021; Tsai and Schmidt 2020; Bailey et al. 2023; Chen et al. 2021). Most importantly, Tsai and Schmidt (2020) profiled the responses of roots of Fe‐deficient Arabidopsis seedlings (reference accession Col‐0) to elevated pH. Moreover, Pérez‐Martin et al. (2021) compared the responses of a carbonate‐tolerant Arabidopsis accession originating from an inland calcareous (A1_c+_) soil and a carbonate‐sensitive accession from a saline‐siliceous coastal soil (T6_c−_) to high pH and high pH combined with bicarbonate exposure (Terés et al. 2019; Busoms et al. 2023). Yet, the transcriptomic responses to the two individually applied stimuli of Fe deficiency and high pH, as well as their combination, have not been analyzed together in any single comprehensive study to date.

Generally, *A. thaliana* is a moderately calcifuge species; thus, it is thought to generally avoid calcareous soils (Terés et al. 2019; Tsai and Schmidt 2021). Among the closest relatives of the short‐lived selfing species *A. thaliana*, *Arabidopsis halleri* (formerly *Cardaminopsis halleri*) is a diploid stoloniferous herbaceous perennial and an obligate outcrosser (Koch and Matschinger 2007; Krämer 2015). As a metal hyperaccumulator and extremophile colonizer of highly heavy metal‐contaminated soils, the species has an exceptionally large edaphic and ionomic range (Stein et al. 2017; Krämer 2010). A field survey of approximately 1972 *A. halleri* individuals at 165 natural sites, of which 59 metalliferous sites contained toxic levels of heavy metals and 106 sites were non‐metalliferous, reported rhizosphere soil pH values between 2.5 and 7.7 (measured in CaCl_2_). *A. halleri* thus offers a chance to study within‐species adaptations to local soil pH in a plant that has a contrasting natural history representative of a wide range of plant species, while ideally suited for the transfer of knowledge from, and for comparisons with, *A. thaliana* (Krämer 2015).

Here, we employ a comprehensive set of controlled hydroponic experimental treatments for analyzing the responses of plants to Fe deficiency and alkaline pH, two central stimuli encountered on calcareous soils, in a reductionist approach. First, we conducted transcriptomics in roots of the widely used *A. thaliana* reference accession Col‐0 to validate our conditions based on existing partial knowledge and to fill critical knowledge gaps alongside. Importantly, this allowed us to identify responses to Fe deficiency in the context of both an ordinary pH (pH 5.5) and an alkaline pH (pH 7.5), alongside comparisons between the different pH levels. In the next step, we addressed the suitability of the same set of experimental treatments for addressing putative parallel evolutionary physiological adaptations to soil pH in *A. halleri*. For this, we comparatively quantified several physiological and morphological responses across accessions of both *A. halleri* and *A. thaliana* originating from contrasting environments, thereby also expanding the reference framework in *A. thaliana* under the conditions of this study. In summary, we provide novel insights into Fe deficiency regulatory networks in *A. thaliana*, and we establish a basis for future in‐depth comparative transcriptomic and mechanistic studies addressing ecological adaptation to local soil pH in *A. halleri*.

## 2 Results

To test conditions for dissecting central characteristic stimuli encountered by plants on calcareous soils, we designed an experiment encompassing exposure to Fe deficiency and alkaline pH alone and in combination, compared to a control treatment with sufficient Fe at an optimal pH. We sequenced the root transcriptomes of 35‐day‐old *A. thaliana* (Col‐0) cultivated hydroponically under control conditions (10 µM FeHBED, pH 5.5) and exposed to Fe deficiency (0.1 µM FeHBED), alkaline pH (pH 7.5), and their combination, for 5 days. The synthetic Fe chelator molecule HBED is comparably specific for Fe^3+^ and prevents the precipitation of Fe^III^ in a solution even at alkaline pH, thus keeping Fe bioavailable, different from other widely used Fe chelators, such as EDTA and poorly standardized mixtures of EDDHA isomers that differ in chemical stability and Fe^3+^ affinity (Chaney 1988; Bin et al. 2016). The Fe deficiency single treatment (L Fe pH 5.5) of this study resembles treatments widely used in the literature to analyze Fe deficiency responses, which are addressed as the ferrome (Mcinturf et al. 2022; Schmidt and Buckhout 2011). Our combined treatment of Fe deficiency with elevated pH (L Fe pH 7.5) resembles the condition which has been used by another transcriptomic study in comparison to a reference treatment of Fe deficiency at optimum pH (pH 5.5) (Tsai and Schmidt 2020). We expect that our combined treatment approximates Fe availability and pH conditions commonly encountered by plants in calcareous soils. Rather than simulating field soil environments, the primary purpose of the Fe‐deficiency single treatment (L Fe pH 5.5) and of the alkaline‐pH single treatment (W Fe pH 7.5) was to gain reductionist insights into the responses to both of these stimuli in isolation, and to compare these with the responses to both stimuli in combination (L Fe pH 7.5). In addition, we expect that the single treatments of this study approximate the conditions naturally encountered by roots locally or temporarily within their rhizosphere in calcareous alkaline soils and possibly elsewhere.

**Figure 1:**
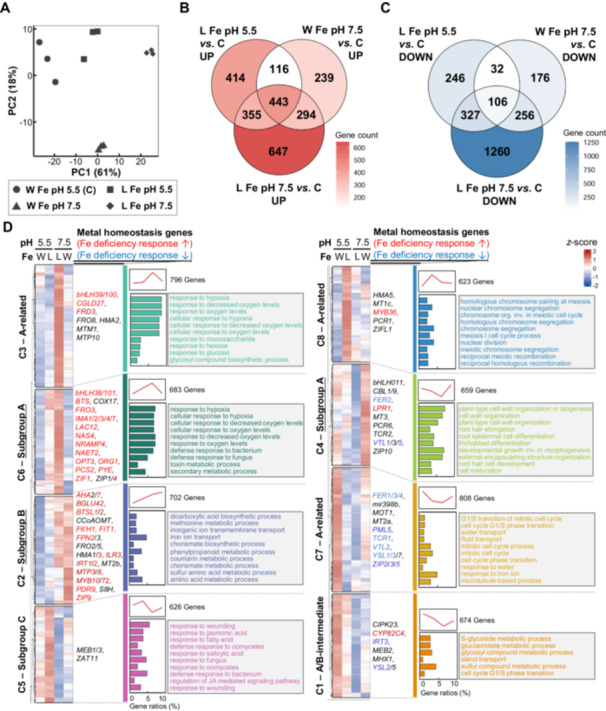
Transcriptomic responses to low‐Fe and high‐pH growth conditions in roots of *Arabidopsis thaliana*. (A–C) Principal component analysis (PCA) showing PC1 and PC2 (A, based on the top 500 most variable transcripts after variance‐stabilizing transformation; see Data S1), and Venn diagrams showing the numbers of transcripts of which the abundance increased (red, B) or decreased (blue, C) after 5 days of exposure to the single low‐Fe treatment (L Fe pH 5.5), the single high‐pH treatment (with iron, W Fe pH 7.5) and their combination (L Fe pH 7.5), compared to the control treatment (W Fe pH 5.5). (D) Clustering of transcriptomic responses (see Data S2). Heatmaps generated by Mfuzz soft clustering of 5571 differentially abundant transcripts, with the names of selected metal homoeostasis‐related genes listed for each cluster (left), the total number of transcripts, a line plot illustrating the representative expression pattern, as well as the top 6–10 significantly enriched gene ontology (GO) biological processes alongside bargraphs showing the fraction of genes in the cluster that are mapped to each GO term (gene ratios) (right) (see Data S3). Highlighted Fe deficiency responses are from this study (light font colours) and from earlier studies (dark font colours). Differential abundance (B–D): |Log_2_(fold change)| > Log_2_(1.5) ≈ 0.585 and adjusted *p* value < 0.05 (*n* = 3 independent experiments, with one pool of tissues from six replicate *A. thaliana* (Col‐0) plants per treatment per experiment). W Fe: 10 μM FeHBED; L Fe: 0.1 μM FeHBED.

A principal component analysis (PCA) clustered the root transcriptomes of three independent experiments by treatment, in accordance with expectations (Figure 1A and Figure S1A,B). PC1 explained 61% of the variance and separated the controls from both single treatments and even more from the combined treatment (L Fe pH 7.5). *bHLH100*, *OPT3, SH8* and *bHLH038* represented notable Fe‐acquisition‐related functions among the 10 transcripts with the highest positive loadings, and *CYP82C4* and *VTL5* among the 10 transcripts with the highest negative loadings, in PC1 (Data S1). PC2 explained 18% of the variance and mostly separated the alkaline‐pH single treatment (W Fe pH 7.5) from all others. Top positive loadings in PC2 were found for *OPT3*, and top negative loadings for *VTL5*, *S8H* and *BGLU42* among the well‐known Fe acquisition‐related genes. PC3 explained 11% of the variance and mostly separated the Fe deficiency single treatment (L Fe pH 5.5) from all others, with top positive loadings for *FSD1* and top negative loadings for the chloroplast‐encoded *psbA* (photosystem II protein D1) and *rbcL* (ribulose‐1,5‐bisphosphate carboxylase/oxygenase), *IRT1*, *CYP82C4* and *FRO2* transcripts, among well‐studied Fe‐related and other notable transcripts. We interpret PC1 to reflect the predominant additive components of the responses to the two single treatments in the response to the combined treatment. By contrast, PC2 and PC3 pinpoint unique aspects of the responses to the single treatments of elevated pH (with bioavailable Fe) and Fe deficiency (at ordinary pH 5.5), respectively, compared to controls, which are not triggered by the combined low‐Fe alkaline‐pH treatment. These unique aspects could reflect plasticity capable of accommodating localized or temporary requirements in a calcareous soil, for example. Alternatively, or in addition, plant responses triggered by the Fe‐deficiency and the alkaline‐pH stimulus alone could poise the plant for an anticipated combined, naturally widespread, low‐Fe alkaline‐pH condition, but then lead to a homoeostatic mal‐adjustment that gives rise to secondary transcriptomic stress responses contributing to PC2 and PC3.

Overall, the transcriptomic response to the combined treatment differed notably from a combination of the responses to the two single treatments. Out of all 1681 genes undergoing an upregulation of transcript levels in response to any single low‐Fe or alkaline‐pH treatment, 59% were shared with the combined treatment, and an additional 35% were unique to the combined treatment (Figure 1B). Compared to the 1143 genes undergoing a downregulation of transcript levels in response to any single condition, 60% were shared by, and an additional 110% were uniquely present in, the combined low‐Fe alkaline‐pH treatment (Figure 1C, |Log_2_FC | ≥ Log_2_(1.5) ≈ 0.585, adjusted *p* value < 0.05). Results of RT‐qPCR‐based quantification of the relative abundance of exemplary transcripts were in very good agreement with these data (Figure S1C–E).

We employed soft clustering (Kumar and Futschik 2007) to group a total of 5571 differentially expressed genes into eight clusters (Figure 1D; any condition vs. control or combined vs. single alkaline‐pH and low‐Fe; differential expression: |Log_2_FC | ≥ Log_2_(1.5) ≈ 0.585, adjusted *p* value < 0.05). Most notably, clustering subdivided the well‐known transcriptional Fe deficiency response (the ferrome), which is reflected here in the comparison between the Fe deficiency single treatment (L Fe pH 5.5) and the control (W Fe pH 5.5), into two differently responding subgroups of transcripts. Transcripts in clusters C4 and 6 show a co‐directional Fe deficiency response under both ordinary‐ and alkaline‐pH conditions (Figure 1D, Data S2), and we thus address this subgroup as ‘pervasively Fe‐deficiency‐responsive’ (pIDR, Subgroup A). The pIDR Subgroup A includes *bHLH38/101*, *BTS*, *IMA1/2/3/4/7*, *OPT3*, *ZIF1* and *PYE*, among others, many of which were previously identified as target genes of the PYE transcription factor (Data S2, see also Section 3) (Long et al. 2010; Schmidt and Buckhout 2011). By contrast, transcripts in cluster C2 show a pattern of increasing levels under Fe deficiency at optimal pH and a co‐directional, strong response to the single alkaline‐pH treatment (i.e., W Fe pH 7.5 vs. control), with no Fe deficiency‐induced increase in transcript levels in alkaline‐pH conditions (i.e., in L Fe pH 7.5 compared to W Fe pH 7.5). We thus term this subgroup ‘additionally alkaline‐pH‐responsive’ (aAPR, Subgroup B). The aAPR Subgroup B includes *IRT1, ILR3/bHLH105, AHA7*, *BTSL1/2*, *F6'H1*, *FIT1 (FIT)*, *MYB10/72*, *FPN2* and *PDR9*, for example, many of which were previously identified as target genes of the FIT transcription factor (Data S2, see also Section 3) (Colangelo and Guerinot 2004; Schmidt and Buckhout 2011). Because soft clustering allows for some heterogeneity of expression patterns within clusters, we designed a set of filters based on these observations to generate clear‐cut subgroup assignments of transcripts (see Data S2 and associated ReadMe sheet).

Cluster C6 contains Subgroup A pIDR transcripts that increased in abundance under Fe deficiency. It shows overall GO term enrichment in hypoxia and oxygen responses as well as anti‐bacterial and anti‐fungal defences, the latter also supported by enrichment of the KEGG term ‘plant–pathogen interaction’ (Figure 1D and Figure S2, Data S2, enrichments listed comprehensively in Data S3 and S4). The second cluster contributing Subgroup A pIDR genes, of which transcript levels decreased under Fe deficiency, cluster C4, includes *FER2* (Reyt et al. 2015), *VTL1* and *TCR2*, with enrichment in cell wall and root hair development‐related GO terms (Data S2). In addition to containing the Subgroup B aAPR genes, cluster C2 shows GO term enrichment for ‘dicarboxylic acid biosynthesis’, ‘methionine metabolism’, and ‘inorganic’ and ‘iron ion transport’, among several others, in general agreement with enrichment in ‘biosynthesis of amino acids’, ‘biosynthesis of aromatic amino acids’ and ‘cysteine and methionine metabolism’ KEGG terms. Cluster C2 also showed enrichment for the KEGG term ‘plant hormone signal transduction’, with transcripts encoding ethylene receptors ERS and ERS2, the phytosulfokine 1 precursor (ATPSK1), cytokinin signalling protein AHP1 as well as several auxin‐related and a few ABA‐related transcripts (Data S4).

Subgroup A‐related clusters C3 and C7 comprise transcripts up‐ and downregulated in abundance under Fe deficiency compared to normal Fe supply, respectively, with a more pronounced response to L Fe pH 7.5 than to L Fe pH 5.5 compared to control conditions. Cluster C3 includes known Fe deficiency‐responsive Fe‐related genes, such as *bHLH39*, *bHLH100* and *CGLD27* (C3), with an overall enrichment in hypoxia‐ and oxygen response GO terms, similar to cluster C6 (see above), and additionally in hexose sugar response‐related GO terms consistent with an enrichment of central metabolism‐related KEGG terms. Cluster C7 comprises Fe deficiency‐suppressed transcripts such as *FER1*/*3*/*4*, *TCR1*, *VTL3*, with an overall enrichment in mitosis and cell cycle‐, as well as iron‐response and water transport‐related GO terms and sulphur‐related KEGG terms. Transcripts in Subgroup A‐related cluster C8 showed a pronounced increase in Fe‐deficient plants only at optimal pH, but not at high pH, and include *MYB36* as a known regulator of Casparian strip formation, which, in turn, influences shoot Fe levels (Kamiya et al. 2015; Whitt et al. 2020). Cluster C8 genes are enriched in meiosis and homologous recombination‐related GO terms, which comprise genes that also act in DNA repair.

Transcripts that were largely downregulated in abundance under all treatment conditions relative to controls, and the most strongly suppressed upon combined low‐Fe alkaline pH treatment, group in cluster C1, which is significantly enriched in genes related to ‘glucosinolate metabolic process’ and related GO terms, consistent with their suggested repression by PYE and ILR3 (Samira et al. 2018). This cluster also contained *CYP82C4* encoding a cytochrome P450 enzyme which modulates Fe acquisition by mediating the conversion of fraxetin, which predominates under high‐pH conditions, to sideretin which predominates in low and intermediate pH (see Section 3) (Paffrath et al. 2024; Rajniak et al. 2018). Finally, the overall expression profile of cluster C5 genes was distinct from both Subgroup A and Subgroup B, with generally higher transcript levels at optimal pH and lower transcript levels at alkaline pH. In response to Fe deficiency, a moderate upregulation at low pH contrasted with a moderate downregulation of transcript levels at alkaline pH. Lacking well‐characterized Fe deficiency‐responsive Fe acquisition genes, cluster C5 is enriched in genes of GO terms related to wounding, jasmonic acid, salicylic acid, fungus and oomycete responses, similar to GO terms enriched in cluster C6, as well as in the KEGG term ‘sesquiterpenoid and triterpenoid biosynthesis’. In summary, the set of treatments of this study identified the distinct regulatory wiring of pIDR and aAPR Fe deficiency response genes within the ferrome and beyond. The multi‐faceted expression profiles implicate a range of biological processes (see Section 3).

**Figure 2:**
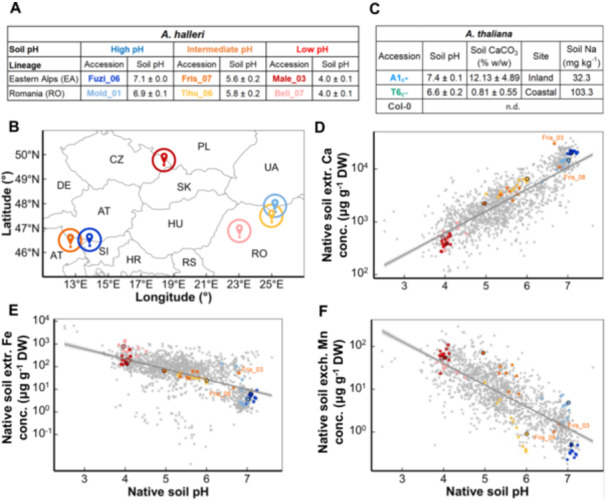
Geographic and soil information for the sites of origin of the *Arabidopsis halleri* and *Arabidopsis thaliana* accessions used here. (A) *A. halleri* accessions (abbreviation of population name, followed by number identifying the plant individual) and pH of rhizosphere soil at the sites of their origin. Soil pH given as arithmetic mean ± SD (*n* ~ 12 plant individuals per site; Stein et al. 2017), measured in 0.01 M CaCl_2_. (B) Map showing geographic origin of *A. halleri* accessions (colours defined in (A)). (C) *A. thaliana* accessions and soil pH, soil CaCO_3_ and soil Na at their sites of origin. Data are arithmetic mean ± SD (*n* = 9 replicate soil samples per site, soil pH measured in 18 MΩ H_2_O) (Terés et al. 2019; Pérez‐Martin et al. 2021). (D–F) Linear correlations with rhizosphere soil pH of soil extractable (in 0.1 M HCl) Ca concentrations (D), soil extractable Fe concentrations (E) and soil exchangeable (in 0.01 M BaCl_2_) Mn concentrations (F) across all non‐metalliferous sites of origin of *A. halleri* (colours defined in (A); individuals specified in (A) are highlighted by a black line around the corresponding symbols). Each data point represents one *A. halleri* individual (*n* = 1466; Stein et al. 2017), and linear regression models are given in Table S1 (D–F). c^+^, calcareous soil origin; c^−^, non‐calcareous soil origin; DW, dry mass; n.d., no data.

We turned to publicly available data for examining the range of pH values in non‐metalliferous soils, that is, not contaminated with elevated levels of heavy metals, hosting natural populations of *A. halleri*. The pH of rhizosphere soil samples, measured in CaCl_2_, spanned values from 2.5 up to 7.6 (median 5.4; site median between 3.9 and 7.1, median among all sites 5.3) (Stein et al. 2017). We chose a set of accessions available in our collection and originating from soils of contrasting pH—low, intermediate and high pH—in two distinct geographic regions and phylogenetic lineages, Eastern Alps (EA) and Romania (RO) (Figure 2A and B) (Pauwels et al. 2012; Koch and Matschinger 2007). By comparison, two previously characterized *A. thaliana* accessions originating from an inland calcareous (A1_c+_) and a coastal non‐calcareous (T6_c−_) soil, reflected smaller differences in soil pH (Figure 2C). Note that the pH of the soils of origin of *A. thaliana* was measured in water, which generally yields results that are approximately 0.6 pH units above soil pH values obtained in 0.01 M CaCl_2_ solution used in the work on *A. halleri* (Schachtschabel et al. 1992; Stein et al. 2017). Across non‐metalliferous field sites of *A. halleri*, there was a positive correlation between soil pH and both HCl‐extractable and BaCl_2_‐exchangeable soil Ca concentrations, supporting the role of CaCO_3_ in soil alkalinity (Figure 2D and Figure S3A, Table S1). Fe concentrations in the extractable fraction spanned more than five orders of magnitude in total, and they were negatively correlated with soil pH (Figure 2E). Leaf Fe concentrations of plants sampled in the field showed little dependence on soil pH (Figure S3B), resulting in a strong positive correlation of leaf Fe:soil extractable Fe ratios with soil pH (Figure S3C). These observations are in agreement with an enhanced efficiency of Fe acquisition in *A. halleri* genotypes inhabiting high‐pH soils in the field. As known, there was a negative correlation of soil exchangeable Mn with soil pH (Figure 2F) (Stein et al. 2017). Ratios of leaf Mn concentrations to soil exchangeable Mn concentrations were positively correlated with soil pH; yet, there was a net decrease of leaf Mn concentrations with increasing soil pH, different from Fe (Figure S3D,E). This suggests that, overall, *A. halleri* plants originating from higher‐pH soils have enhanced capacities to extract Fe and also to take up bioavailable Mn from the soil and accumulate these nutrients in their leaves. With respect to the soil pH‐related parameters analyzed here, the chosen accessions (see Figure 2A) were representative of the *A. halleri* genotypes collected at the same field site, and populations on soils of similar pH showed similar properties in the field in the different geographic regions. As a minor difference by comparison, the soil data suggested elevated available levels of Mn and S, and a smaller excess of available Ca, in Romanian compared to Eastern Alpine high‐pH soil samples (Figure 2F and Figure S3A,F–K).

**Figure 3:**
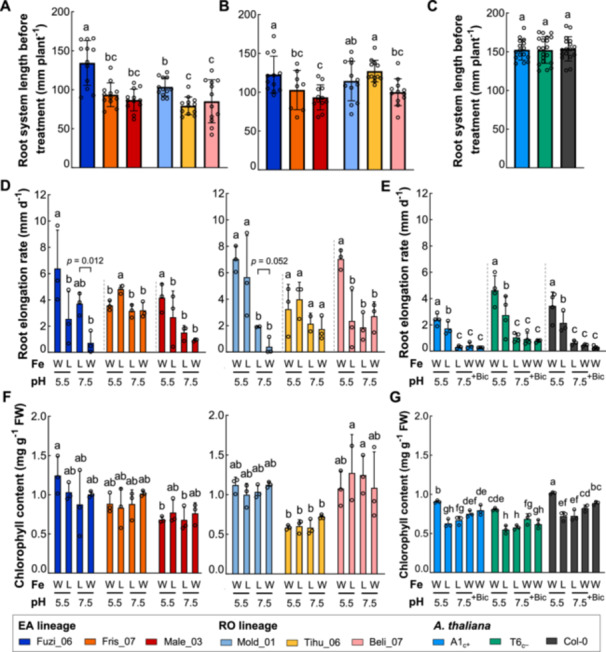
Root growth and leaf chlorophyll content in different accessions of *Arabidopsis halleri* and *Arabidopsis thaliana*. (A–C) Total lengths of the root systems of 4‐week‐old *A. halleri* ((A, B); each corresponding to an independent experiment) and 5‐week‐old *A. thaliana* (C) plants upon pre‐cultivation in control hydroponic solutions prior to treatment. (D–G) Average root elongation rate of the longest root per plant (D, E) and leaf chlorophyll contents (F, G) of *A. halleri* (D, F) and *A. thaliana* (E, G) measured after exposure to low‐Fe and high‐pH conditions alone and in combination for 5 d. Bars show arithmetic mean ± SD of *n* = 8–12 replicate clones (A, B), *n* = 16–20 replicate plants (C), *n* = 3 replicate clones (D) and plants (E), *n* = 3 replicate clones (F), and *n* = 3 independent experiments (G), based on one arithmetic mean of three replicate plants per experiment. Distinct letters denote statistically significant differences (*p* < 0.05) according to ANOVA (Duncan's test), analyses across groups (separated by vertical dashed grey lines), as follows: all *A. halleri* (A, B), all *A. thaliana* (C); each accession of *A. halleri* (D), *A. thaliana* (E); all accessions per lineage of *A. halleri* (F), all *A. thaliana* accessions (G). For specific pairwise comparisons, Student's *t*‐test was used, with the resulting *p* values given inside the diagrams. Data shown in (D) are from one experiment representative of two independent experiments (results of another independent experiment shown in Figure S5C). +Bic, supplemented with 10 mM bicarbonate (NaHCO_3_); for other information see Figures 1 and 2.

For a basal characterization under the standardized cultivation conditions established in this study (see Figure 1), we cultivated the chosen set of six *A. halleri* accessions (see Figure 2A), alongside *A. thaliana* accessions Col‐0, A1_c+_ and T6_c−_ (see Figure 2C) for reference. Following pre‐cultivation in a standard (control) hydroponic solution, roots of *A. halleri* accessions originating from the high‐pH soils were longer than roots of *A. halleri* accessions originating from low‐pH soils (Figure 3A–C and Figure S4; statistically significant at *p* < 0.05 for EA in both independent experiments, and for RO in the first independent experiment). The shorter root systems of low‐pH accessions appeared wider and denser on photographs, suggesting a more strongly branched pattern than in other *A. halleri* accessions (Figure S4; in all cases except RO in the first experiment). By contrast, the root lengths of all *A. thaliana* accessions A1_c+_, T6_c−_ and Col‐0 were uniform at the end of the pre‐cultivation period (Figure 3C and Figure S4J–L). After 5 days of exposure to low‐Fe and alkaline‐pH treatment as established in *A. thaliana* (Col‐0) (see Figure 1 and Figures S1 and S2), we measured root lengths and calculated average root elongation rates. Root elongation rates in *A. halleri* originating from high‐pH soils were very strongly decreased following exposure to elevated pH with available Fe when compared to controls, convergently in the EA and RO lineages (Figure 3D,E and Figure S5A–D; statistically significant at *p* < 0.05 throughout). In both accessions originating from low‐pH soils, exposure to low Fe convergently resulted in decreased root elongation at both ordinary and elevated pH, and largely also at elevated pH with sufficient Fe (except RO in Figure S5D), when compared to controls (Figure 3D,E and Figure S5A–D; statistically significant at *p* < 0.05 throughout). Upon cultivation in low Fe at pH 5.5, root growth of the low‐pH accession Beli_07 was significantly impaired, in contrast to the high‐pH accession Mold_01, within the RO lineage (Figure 3D and Figure S5A,C,D). In all *A. thaliana* accessions, we observed moderately decreased root elongation in response to low‐Fe conditions at pH 5.5 and a further, more pronounced decrease in root elongation in alkaline‐pH conditions irrespective of Fe supply, as well as following treatment with 10 mM sodium bicarbonate (Figure 3E and Figure S5B). None of the treatments had a significant effect on leaf chlorophyll concentrations in any of the *A. halleri* accessions (Figure 3F and Figure S5E). However, in *A. thaliana*, all low‐Fe, alkaline‐pH and bicarbonate treatments resulted in significantly lowered leaf chlorophyll contents across all accessions, with a general trend for smaller effects in the treatments including bioavailable Fe (Figure 3G and Figure S5F). Accession A1_c+_ maintained higher chlorophyll contents than T6_c−_ upon cultivation in low Fe at pH 7.5 and also with bicarbonate, consistent with expectations (Terés et al. 2019), and Col‐0 maintained higher chlorophyll contents than A1_c+_ upon cultivation in low Fe at ordinary pH and in the bicarbonate treatment. In the control cultivation condition employed, chlorophyll contents were highest in Col‐0, lower in A1_c+_ and lowest in T6_c−_, which complicates comparisons among *A. thaliana* accessions.

**Figure 4:**
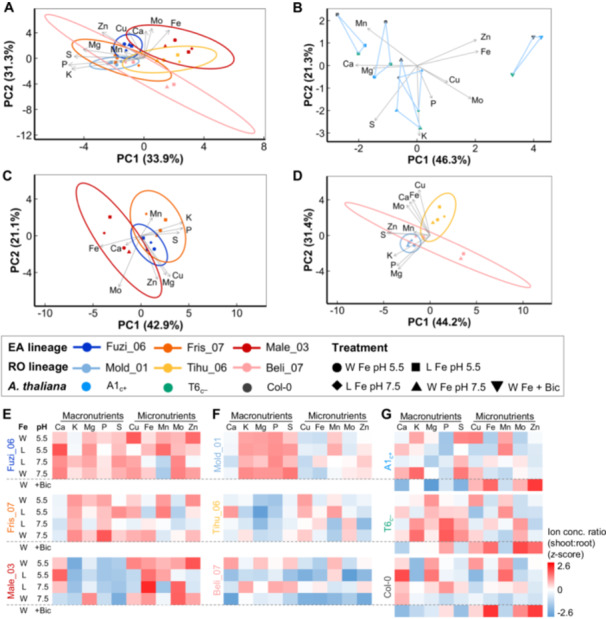
Shoot‐to‐root ionome ratios of *Arabidopsis halleri* and *Arabidopsis thaliana* grown in hydroponics varying Fe supply and pH. (A–D) Principal component analysis (PCA) biplots of shoot:root ratios of concentrations of each element in *A. halleri* (A), *A. thaliana* (B), and for the subsets of the Eastern Alps (C) and Romania (D) lineage of *A. halleri*. Ellipses show 95% confidence limits around group centroids. Lines forming light blue triangles connect data points from different genotypes for a common treatment (B, shown for visual guidance). Arrows indicate the loadings of elements contributing to PC1 and PC2. (E–G) Heatmaps of elemental concentration ratios (shoot:root) in the EA (E) and RO (F) lineages of *A. halleri* and in *A. thaliana* (G). PCA and heatmaps are based on *z*‐scores calculated separately for each species from Log_10_‐transformed concentration ratios (Log_10_[(shoot elemental conc.)/(root elemental conc.)]). Shown are arithmetic means (*n* = 3 replicate clones for *A. halleri* and *n* = 3 independent experiments for *A. thaliana*, each represented by the mean of three replicate plants). W Fe, control Fe (10 μM FeHBED); L Fe, low Fe (0.1 μM FeHBED); c^+^, from calcareous soil; c^−^, from non‐calcareous soil; EA, Eastern Alps; RO, Romania; +Bic, supplemented with 10 mM bicarbonate (NaHCO_3_); for other treatments and designations, see Figures 1 and 2.

We used ICP‐OES to conduct multi‐element analyses of root and shoot tissues for all plants after treatment and final harvest. PCA grouped the ionomes of *A. halleri* by accession, but those of *A. thaliana* by treatment instead, for shoots and roots, as well as for shoot:root ion concentration ratios (Figures S6A–C, S7A–C and Figure 4A–D). Shoot:root elemental concentration ratios were the most stable across treatments in *A. halleri* accessions originating from high‐pH soils and more treatment‐dependent in *A. halleri* accessions originating from low‐pH soils, and the most treatment‐dependent in *A. thaliana* (Figure 4A–G). This was predominantly a result of less treatment‐dependent and higher macronutrient ion concentrations in shoots across conditions in the high‐pH accession Mold_01 compared to the low‐pH accession Beli_07 in the RO lineage (Figure S6B,E). In the EA lineage, shoot:root elemental concentration ratios were more strongly influenced by more stable and lower macronutrient ion concentrations in roots of the high‐pH than of the low‐pH accession (Figure S7A,D). Between‐species comparisons revealed that shoot:root macronutrient element concentration ratios were generally lower in *A. halleri* than in *A. thaliana* (Figure S8A). The predominant contributions to cross‐species ionomic differences were made by lower concentrations of P and K, but higher concentrations of Fe and Zn in shoots of *A. halleri*, and lower concentrations of Zn, Fe and Mo in roots of *A. halleri*, compared to *A. thaliana* (Figure S8B,C).

**Figure 5:**
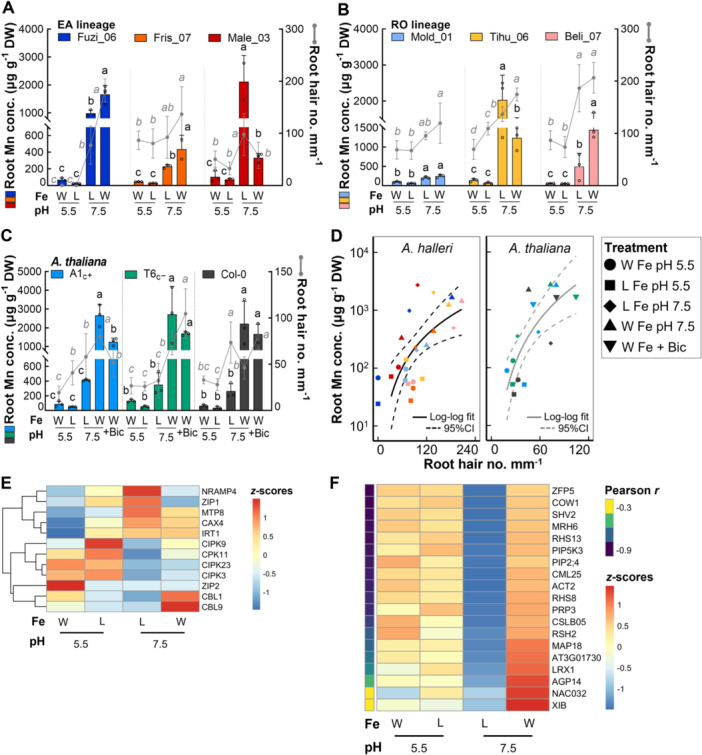
Concentrations of Mn in roots and root hair density in *Arabidopsis halleri* and *Arabidopsis thaliana* grown in hydroponics varying Fe supply and pH. (A–C) Dual bar‐and‐line graphs showing root Mn concentration (left vertical axis, bars) and root hair density (right vertical axis, lines) in the EA (A) and RO (B) lineages of *A. halleri* and in *A. thaliana* (C). Root hair density is the number of root hairs counted within a segment of 0.5 mm in length upward from the onset of the differentiation zone near the root apex. Shown are means ± SD (for concentrations, *n* = 3 replicate clones for *A. halleri* and independent experiments for *A. thaliana*, each represented by the mean of three replicate plants; for root hair density, *n* = 4–6 longest roots randomly selected among three replicate clones of *A. halleri*, and *n* = 3–4 replicate plants of *A. thaliana*). (D) Scatter plots showing arithmetic means and regressions reflecting the relationships between root hair density and root Mn concentrations in *A. halleri* (left) and *A. thaliana* (right; from data as shown in A‐C). Regressions (solid black lines, fit) and 95% confidence intervals (dashed lines, CIs) were fitted on Log_10_‐transformed data after excluding all samples with zero root hairs (both data points for Fuzi_06 at pH 5.5), and subsequently back‐transformed for plotting (vertical axes Log_10_‐scaled, all data points included in the plot; see Table S2). (E, F) Transcript levels of genes encoding transmembrane Mn transporter proteins (E) and root hair development and growth‐related proteins (F) from transcriptome sequencing of *A. thaliana* (Col‐0, see Figure 1). Pearson correlation coefficients (*r*) in (F) are given for the relationship between Log_2_‐transformed normalized transcript levels and root hair density (see (C)) in *A. thaliana* Col‐0 (based on one arithmetic mean per treatment). Distinct characters denote statistically significant differences (*p* < 0.05) according to ANOVA (Duncan's test; A–C). W Fe, control Fe (10 μM FeHBED); L Fe, low Fe (0.1 μM FeHBED); +Bic, bicarbonate; c^+^, from calcareous soil; c^−^, from non‐calcareous soil; EA, Eastern Alps; RO, Romania.

We noted differences in root hair density between treatments in both *A. halleri* and *A. thaliana* plants of the same genotype. Generally, root hair densities (see Materials and Methods) were increased in plants upon growth in high‐pH media (Figure 5A–C and Figure S9A–C). In addition, root hair densities were higher in bicarbonate‐treated plants compared to plants cultivated at pH 7.5 in *A. thaliana* T6_c−_, but not in A1_c+_ originating from a calcareous soil, and not in Col‐0. Other quantitative differences between accessions with respect to root hair density in ordinary pH (5.5), in elevated pH (7.5 or bicarbonate treatments), or their responses to pH or Fe showed no evident relationships with the pH of the soil of plant origin (Figure 5A–C and Figure S9A–C). There was a positive correlation between root hair densities and Mn concentrations in roots of both species (Figure 5A–D and Figure S10, Table S2). Out of the transcripts encoding Mn homoeostasis‐related genes (Meier et al. 2025), the abundances of *MTP8*, *NRAMP4*, *CAX4*, *IRT1*, *CBL9* (positive), as well as *CPK11*, *CIPK3* and *CIPK23* (inverse) showed profiles similar to those of root hair densities and Mn concentrations in Col‐0 (compare Figure 5C,E, see also Figure 1). *IRT1*, *CAX4* and *MTP8* can be considered as candidate genes for contributing directly to root Mn accumulation at alkaline pH. In addition, the abundance of root hair‐related transcripts responded to elevated pH and low Fe (Figure 5F, cluster C4 in Figure 1; Data S2 and S3). In general, transcript levels of these genes in *A. thaliana* (Col‐0) were the lowest upon combined Fe deficiency and alkaline‐pH treatment and the highest in the alkaline‐pH single treatment, compared to controls (Figure 5F). Transcript levels of most of the root hair‐associated genes in cluster C4 were negatively correlated with root hair density across the four conditions (Figure 5F, Data S5; albeit not statistically significant because of the low number of four datapoints per gene). The hydroponic cultivation system employed because of a lack of seeds of *A. halleri* precluded the reliable quantification of root hair lengths, which tended to increase upon cultivation at alkaline pH and appeared maximal in the presence of available Fe in Col‐0 and most other genotypes (Figure S9C; see also Section 3).

Root hair densities showed no correlation with shoot Mn concentrations or with root or shoot Fe concentrations in either *A. halleri* or *A. thaliana* or any subset of accessions (Figure S10A–I). In all *A. thaliana* accessions, shoot Fe concentrations qualitatively mirrored Fe supply in the hydroponic medium (Figure S10C). Compared to controls, Fe concentrations in *A. thaliana* roots were significantly lower upon alkaline‐pH treatment with available Fe and further reduced in bicarbonate‐treated plants, thus suggesting lowered Fe immobilization in roots under these conditions. Shoot Mn concentrations were about 1.5‐fold elevated in plants cultivated at alkaline pH with available Fe (W Fe pH 7.5) compared to all other cultivation conditions in *A. thaliana* A1_c+_ and T6_c−_, consistent with a reinforced partitioning of Mn into shoots in this condition (Figure S10I). The profiles of shoot and root Fe and Mn concentrations in *A. halleri* differed among accessions, with an overall trend for lower Fe levels and higher Mn levels in plants subjected to single and combined treatments by comparison to controls.

Besides a positive correlation of root hair number with root Mn concentrations, we observed a negative correlation with root Mo concentrations in *A. thaliana* (Figure S11A). This implies a negative correlation between Mo and Mn concentrations in roots, which we observed in both *A. thaliana* and *A. halleri* (Figure S11B). In soil, Mo availability is known to increase with increasing pH, opposite to Mn availability. Additionally, the expression profile of *MOT1* encoding the root molybdate (MoO_4_^2−^) uptake system (Tomatsu et al. 2007) was approximately inverse to the expression profile of *MTP8* in *A. thaliana* (Col‐0). Compared to controls, the transcript levels of *MOT1* were decreased upon growth at alkaline pH and low Fe, with additive effects (Figure S11C, Data S2).

## 3 Discussion

Calcareous soils have a strong impact on plant ecology and agriculture in the field, yet the present knowledge of how plants cope with or adapt to such habitats remains insufficient (Bontpart et al. 2024; Poormohammad Kiani et al. 2012; Tsai and Schmidt 2021; Pérez‐Martin et al. 2024; Maron et al. 2013). Here we first tested a set of four hydroponic cultivation conditions designed to dissect transcriptional responses to central properties of calcareous soils, low bioavailable Fe levels and alkaline pH, in the reference accession *A. thaliana* (Col‐0). We then identified and characterized six natural *A. halleri* accessions originating from low, intermediate and high‐pH soils in two geographically separated regions that are inhabited by distinct phylogenetic lineages (Koch and Matschinger 2007; Pauwels et al. 2012), respectively, based on existing field data. These six *A. halleri* accessions, as well as three *A. thaliana* reference accessions, were then cultivated to comparatively examine the effects of our set of treatments on root elongation, root hair formation, leaf chlorophyll contents and the ionome.

We found that the well‐known root transcriptional Fe deficiency response genes acting in Fe homoeostasis of *A. thaliana* (Col‐0), observed in this study and previously on synthetic media at pH values between 5.5 and 5.8 (optimal range), form two subgroups differing in the responses of transcript levels in alkaline conditions. Compared to the response to Fe deficiency at optimal pH, the pIDR Subgroup (A) responds co‐directionally to Fe deficiency at alkaline pH. This is different from the aAPR Subgroup (B), which instead shows a response of transcript levels to alkaline pH alone (in the presence of bioavailable Fe) that is co‐directional with the Fe deficiency response at optimal pH values. Both subgroups contain genes encoding regulatory as well as downstream effector proteins, giving rise to the expectation that the regulators control the transcript levels of effector genes in the same subgroup.

Proteins encoded by pIDR genes comprise well‐characterized transcription factors bHLH38/101 (Group Ib) and PYE (bHLH47/Group IVb), as well as the regulators BTS and IMA1/2/3/4/7. The effector proteins in the pIDR Subgroup tend to have roles in the within‐plant or intracellular partitioning of Fe or of Fe chelator molecules. This subgroup contains all genes addressed as PYE targets (Schmidt and Buckhout 2011), for example, *NAS4* and *ZIF1* (Long et al. 2010). The aAPR Subgroup B transcription factors are FIT (FIT1, bHLH29, Group IIIa), ILR3 (bHLH105, Group IVc) and MYB10/72, and the regulatory proteins are BTSL1/2. The effector genes in this group are involved in root Fe acquisition, including *IRT1*, or in the detoxification of secondary IRT1 substrates in roots. *IRT1* and *FRO2* are thought to be direct targets of FIT‐mediated regulation (Yuan et al. 2008), and a number of aAPR effector genes are proposed FIT targets, including *IRT2*, *MTP3*, *MTP8*, *HMA3* and *ZIP9*, among others (Connolly et al. 2002; Schmidt and Buckhout 2011). MYB72 regulates *BGLU42* (Zamioudis et al. 2014). In the root differentiation zone, for the majority of Subgroup B (aAPR) genes the highest transcript levels or promoter activities were reported outside the stele in the epidermis, the cortex and/or the endodermis, which are exposed to solutes in the soil solution, including *FIT*, *FRO2*, *AHA7* and *IRT1* (Colangelo and Guerinot 2004; Connolly et al. 2003; Hoffmann et al. 2019; Vert et al. 2002). By comparison, promoter activities or transcript levels of the majority of the Subgroup A (pIDR) genes are the highest in the stele in this root zone, which is shielded from the soil solution by the Casparian strip, including *PYE*, *NAS4*, and *FRD3* (Long et al. 2010; Palmer et al. 2013; Rogers and Guerinot 2002). This was in agreement with the cell type of the greatest Fe deficiency‐responsive change in transcript levels for the vast majority of metal homoeostasis genes in Subgroups A (pIDR) and B (aAPR) (see Figure 1D, Data S2) (Dinneny et al. 2008). Among only few exceptions in their respective Subgroups were *ILR3* transcripts, with the largest Fe deficiency‐responsive change in the stele, and Group Ib *bHLH* gene transcripts, with generally larger changes outside the stele (Dinneny et al. 2008). The identification of the pIDR and aAPR subgroups organizes the hierarchically organized Fe deficiency response regulation into two distinctly responsive parts, which appear to carry information on how shoot‐derived systemic signals and local signals are integrated. In addition to the presence of Fe (Vert et al. 2003), alkaline pH may act similarly as a local cue in the rhizosphere. The comparison of our data with current models suggests that there are also examples of regulation across the Subgroups identified here (Spielmann et al. 2023; Gao et al. 2019): FIT interacts with group Ib bHLH transcription factors in the regulation of Fe uptake, PYE interacts with ILR3 in the regulation of Fe mobilization, and *NAS4* is thought to be a target also of MYB10/72‐mediated regulation (Palmer et al. 2013).

A previous transcriptome study compared Fe‐deficient roots cultivated at pH 7.0 and Fe‐deficient roots cultivated at pH 5.5 (Tsai and Schmidt 2020). Out of the differentially expressed genes, the authors then identified 54 genes for which they had previously published differential expression under Fe deficiency compared to Fe sufficiency at pH 5.5. Among this subset of genes, they observed co‐directional regulation in response to increased pH compared to Fe deficiency for *S8H* and *NAS4*. Our data concur with this. By contrast, Tsai and Schmidt (2020) reported that the vast majority of ferrome genes with functions in Fe‐related metal balancing were anti‐directionally regulated. Out of these, we observed anti‐directional regulation of gene expression between the corresponding conditions only for *FSD1*, that is, 1 out of 11 genes. The data used by Tsai and Schmidt (2020) were from seedlings cultivated on agar‐ or Gelrite‐solidified media containing sucrose, cultivated at a low light intensity and treated from germination on for 14 days in the Fe‐deficient medium at pH 7 including poorly soluble FeCl_3_ (Tsai and Schmidt 2020), or for 3 days before harvest with ferrozine used to induce Fe deficiency at pH 5.5 (Rodriguez‐Celma et al. 2013). Different from Rodriguez‐Celma et al. (2013), the transcript encoding CYP82C4, which converts fraxetin to sideretin, showed no significant response to Fe deficiency at acidic pH in our hydroponic system (Data S2). Alkaline pH caused a strong repression of *CYP82C4* transcript levels, consistent with the pH‐dependent regulation reported in agar‐grown seedlings (Pérez‐Martin et al. 2024; Tsai and Schmidt 2020).

Among 419 genes differentially expressed upon exposure to high pH (pH 7.5, buffered with MOPS) in media containing FeEDDHA for 6 h (Bailey et al. 2023), only three genes (*IREG2*, *MTP8* and *S8H*) overlapped with our aAPR Subgroup delineated here and were previously identified as FIT targets (Schmidt and Buckhout 2011). In addition, one gene (*VTL5*; Bailey et al. 2023) was included in the pIDR Subgroup identified here and overlapped with known PYE‐target genes (Schmidt and Buckhout 2011). Notably, the direction of regulation observed for the levels of these transcripts was consistent with this study. A total of 66 genes overlapped between the DEGs identified in our study and those regulated in response to NaHCO_3_ (3 mM; pH ~ 8) or NaOAc (4 mM; pH ~ 7) treatments for 4 days in media containing FeEDTA (Chen et al. 2021) and previously reported as part of the ferrome (Schmidt and Buckhout 2011). In particular, FIT target aAPR genes (according to this study) were predominantly enriched in the modules M and H, which Chen et al. (2021) identified by WGCNA as highly correlated with and specific to NaHCO_3_‐treated roots, including genes such as *S8H*, *MTP8*, *MYB10*/*72* and *IRT1*. Both of these studies highlighted a broad pH‐associated transcriptional reprogramming including genes related to Fe homoeostasis, but they were not designed to identify the full set of pH‐ and Fe deficiency‐dependent transcriptional responses examined here. All in all, differences in cultivation and treatment conditions as well as in plant age may account for deviating aspects between the results of these previous studies and the work described here. The clustering in this study also identified regulation in functions outside homoeostasis of Fe and other metals, notably for DNA repair, pathogen responses, sugar responses, as well as root hair development and elongation, which should be addressed in the future (for root hairs, see below).

We conducted experiments addressing the effects of the set of treatments established here in *A. thaliana* Col‐0 additionally on six *A. halleri* accessions originating from soils of contrasting pH by comparison to two accessions of *A. thaliana*, which were previously identified to differ in bicarbonate tolerance. Compared to *A. thaliana*, our phenotypic observations of root growth, leaf chlorophyll contents and ionomes suggest a generally enhanced tolerance to Fe deficiency and alkaline‐pH treatments in *A. halleri* as well as a larger within‐species phenotypic divergence in relation to the pH of the soil of origin, consistent with species‐wide and local adaptation (Figures 3, 4, 5 and Figures S4–S8). Longer root systems, as observed after pre‐cultivation in control medium in the *A. halleri* accessions from high‐pH soils, have been related to improved plant performance on calcareous alkaline soil (Lynch et al. 2024). The two *A. halleri* accessions originating from high‐pH soils in the Eastern Alps and Romania lineages, Fuzi_06 and Mold_01, showed some convergent aspects, namely a more stable ionome and shared responses of root growth (Figures 3, 4, 5 and Figures S5–S8) including a strong attenuation of root elongation upon the alkaline‐pH single treatment (W Fe pH 7.5) compared to the controls (W Fe pH 5.5). This may reflect a toxicity symptom caused by excessive Fe uptake resulting from highly effective Fe acquisition operating in these accessions at high pH as an adaptation to local soil pH, taking into account that final bulk tissue Fe concentrations often do not reflect physiological Fe status or the activity of Fe acquisition. Alternatively, this root growth response could reflect an adaptive response that protects roots from excess Fe‐mediated damage when the composition of the soil solution fluctuates on calcareous alkaline soil. These hypotheses will be examined in the future. Element‐specific ionomic differences and other root growth responses may reflect lineage‐specific adaptations to calcareous alkaline soil. Notably, we also observed some differences in soil composition between the Mold and Fuzi field sites (Figure 2 and S3).

In agreement with our expectations based on Terés et al. (2019), *A. thaliana* accession A1_c+_ retained higher chlorophyll contents relative to controls in the bicarbonate treatment than T6_c__−_ (Figure 3G). According to chlorophyll contents, *A. thaliana* Col‐0 was not more sensitive than A1_c+_ to Fe deficiency, alkaline pH and bicarbonate exposure. The comparison of root elongation rates between A1_c+_ and T6_c__−_ was complicated by large between‐accession differences under control conditions. Comparably mild differences between accessions could result from the presence of available Fe in our bicarbonate conditions, in contrast to the earlier study. As an aspect of phenotypic plasticity largely shared across both species and all genotypes, we observed increases in root hair density most pronouncedly under alkaline‐pH conditions, in general agreement with previous observations (Müller and Schmidt 2004). One cluster (C4) of transcripts was enriched in root hair development genes, and their transcript levels changed across conditions in a similar manner to root hair densities. However, root hair‐related transcripts showed minimum abundances upon the combined Fe‐deficiency alkaline‐pH treatment, accompanied by maximum average root hair density in Col‐0 (Figure 5C,D,F). For most of the cluster C4 genes, transcript levels are negatively correlated with root hair density, with *ZFP5* showing the strongest association (Figure 5F, Data S5). Both the number and the lengths of root hairs are reduced in *zfp5* mutants, but the effects of nutrient deficiencies on these phenotypes can be complex and are unknown for Fe deficiency and high pH (An et al. 2012; Huang et al. 2020). *COW1* and *MRH6* regulate distinct aspects of root hair development, including tip growth and polarity (Böhme et al. 2004; de Campos and Schaaf 2017; Jones et al. 2006). This functional and environment‐dependent diversity supports the idea that cluster C4 comprises regulators acting on different aspects of root hair development and elongation, supporting an overall complex relationship between cluster C4 transcript levels and root hair density. Moreover, there are additional genes known to strongly influence root hair development under elevated pH, including *AHA7* and, to a lesser extent, *AHA2* (cluster C2) (Santi and Schmidt 2009). Based on *aha7* mutant phenotypes, *AHA7* function is required for the increase in root hair density under Fe deficiency (Santi and Schmidt 2009). The levels of *AHA7* transcripts, which are comparably root‐hair specific, were the highest in both of our alkaline‐pH treatments (pH 7.5: 7300 in L Fe and 8600 W Fe; pH 5.5: 4200 in low Fe and 1200 W Fe, normalized counts), thus matching root hair density more closely than cluster C4 transcript levels. Root hair initiation and elongation involve localized fast dynamics in apoplastic and cytosolic pH as well as Ca^2+^ and ROS levels (Stéger and Palmgren 2022), with potentially complex interactions in synthetic media buffered at an alkaline pH that will require further study.

At alkaline pH, the oxidation of Mn^2+^ to poorly soluble Mn oxides in soils can restrict Mn availability, and plants respond by promoting root hair development and acidifying the rhizosphere, thereby enhancing Mn mobilization and uptake (Alejandro et al. 2020). In agreement with our observations, Giehl et al. (2023) reported that under conditions restricting Fe bioavailability through a high pH (pH 6.7 vs. pH 5.5; both with 30 µM FeEDTA and 80 µM Mn), root Mn levels increased and Mn selectively accumulated in trichoblasts of Arabidopsis, thereby preventing Fe deficiency‐induced Mn toxicity in the shoot. Notably, when root hair formation was inhibited at pH 6.7, both the Mn content of *pEXP7*‐positive trichoblasts and *MTP8*‐dependent Mn retention in roots were decreased (Giehl et al. 2023). Root Mn and Mo levels were correlated with both medium pH and root hair density, and they were paralleled by transcript levels of Mn homoeostasis genes and *MOT1* (Figures 5 and S11). It is interesting to note that genome‐wide association studies identified an association of *MOT1* with local soil pH in *A. thaliana* and soybean (Poormohammad Kiani et al. 2012; Zhang et al. 2023).

To sum up, our results suggest that in response to conditions encountered by plants on calcareous soil, they orchestrate nuanced and complex processes of physiological, transcriptomic and morphological re‐organization involving two regulatory sub‐networks of the Arabidopsis ferrome. Moreover, we conclude that *A. halleri* is a suitable species for in‐depth genomic and genetic approaches studying plant adaptations to local soil pH, employing the findings of this study as a basis for reference. Once high‐pH adaptations are better understood in *A. halleri* originating from non‐metalliferous soils, it may be insightful to expand the scope of the study by including *A. halleri* originating from metalliferous soils, which tend to exhibit elevated pH values overall (Stein et al. 2017).

## 4 Materials and Methods

### 4.1 Plant Material and Growth Conditions

Seeds of *A. thaliana* (accession Col‐0) were obtained from NASC (https://arabidopsis.info/), and of accessions A1_c+_ and T6_c__−_ from Charlotte Poschenrieder (Universitat Autónoma de Barcelona, Bellaterra, Spain) (Terés et al. 2019). For the pre‐cultivation of *A. thaliana*, seeds were sterilized by immersion in 70% (v/v) ethanol for 1 min and subsequently in 2% (w/v) NaOCl and 0.01% (v/v) Triton X‐100 for 10 min, followed by five washes in sterile ultrapure water. Eight to ten seeds were transferred into one square 120‐mm polystyrene Petri dishes (Greiner Bio‐One GmbH, Frickenhausen, Germany) containing 40 mL of a modified Hoagland's solution (1.5 mM Ca(NO_3_)_2_, 0.28 mM KH_2_PO_4_, 0.75 mM MgSO_4_, 1.25 mM KNO_3_, 25 µM H_3_BO_3_, 5 µM MnSO_4_, 2.5 µM ZnSO_4_, 0.25 µM CuSO_4_, 50 µM KCl and 0.1 µM Na_2_MoO_4_, buffered to pH 5.7 with 3 mM 2‐(*N*‐morpholino)ethanesulfonate [MES]) prepared in ultrapure water ([Becher et al. 2004], with modifications), containing 5 µM FeHBED, a complex prepared from FeCl_3_ and N,N′‐di‐(2‐hydroxybenzoyl)‐ethylenediamine‐N,N′‐diacetate (HBED, Strem Chemicals, Bischheim, France; catalogue number 07‐0422, with a 0.5% excess of HBED over Fe), with 1% (w/v) sucrose and solidified with 0.8% (w/v) agar Type M (Merck KGaA, Darmstadt, Germany). After stratification at 4°C in the dark for 60 h, seedlings were cultivated on vertically oriented plates in a growth chamber (CFL Plant Climatics, Wertingen, Germany) under 11‐h light (22°C, 145 µmol m^−2^ s^−1^ white light) and 13‐h dark (18°C) cycles at 65% relative humidity for 21 days. For the pre‐cultivation of *A. halleri* accessions originating from six natural populations (see Figure 2), cuttings of 15–30 mm in length were taken from plants growing on soil (Minitray soil, Balster Einheitserdewerk, Frӧndenberg, Germany) and obtained through the vegetative propagation of field‐collected plants (Stein et al. 2017). Cuttings were rooted and pre‐cultivated in modified Hoagland's solution containing 5 µM FeHBED at pH 5.7, supplemented with benomyl (Sigma‐Aldrich, St. Louis, MO, USA) at a final concentration of 3 mg L^−1^ (from a 5 mg mL^−1^ stock solution in dimethyl sulfoxide) for 18 days (20 cuttings of a given genotype in each of two containers of 750 mL hydroponic solution). In a second pre‐cultivation step, four vegetative clones of the same genotype were transferred into a 1‐L vessel containing modified Hoagland's solution with 10 µM FeHBED and maintained at pH 5.7 for another 10 days (*A. halleri*) or 14 days (*A. thaliana*). All hydroponic containers were maintained in trays covered with transparent lids.

Three days before initiating the treatments, the transparent lids were removed, three plants of uniform size were retained per 1‐L container, and aeration of the hydroponic solutions was installed and maintained until harvest. To remove apoplastically bound Fe prior to treatment, all roots were washed in a solution containing 0.1 mM EDTA, 1.25 mM KNO_3_, 0.1 mM Ca(NO_3_)_2_ and 3 mM MES (pH 5.7) for 6 min and subsequently transferred to iron‐free modified Hoagland's solution for 20 min, with gentle movement up and down once every 2–3 min. All hydroponic solutions were exchanged twice per week during pre‐cultivation and treatments.

To start with plants of a similar rosette size, 5‐week‐old *A. thaliana* and 4‐week‐old *A. halleri* plants were transferred into the four treatment solutions. Treatments were conducted in modified Hoagland's solution containing 10 and 0.1 µM FeHBED (W Fe and L Fe) at pH 5.5 and 7.5, with all of the solutions containing both 3 mM MES and 10 mM HEPES buffered to the required pH with KOH as needed. Treatment duration was 5 days, with a replacement of treatment solutions on Day 3. For the additional bicarbonate treatment (+Bic) of *A. thaliana*, the modified Hoagland's solution contained 10 µM FeHBED and 10 mM NaHCO_3_ (initial pH 8.3 in fresh solution, final pH 8.6 in spent solution).

For preliminary experiments aimed at testing possible experimental conditions, we pre‐cultivated *A. thaliana* Col‐0 as described above (Figure S12). Treatments were 5 and 0 µM FeHBED at pH 5.5, buffered with 3 mM MES and unbuffered, as well as 5 µM FeHBED at pH 7.5, buffered with 10 mM HEPES and unbuffered, in modified Hoagland's solution for 6 days. After harvest, we quantified shoot and root Mn and Fe levels.

### 4.2 RNA‐Sequencing and Data Analysis

Three independent experiments were conducted, each with two replicate hydroponic vessels per treatment. Root tissues were harvested from six *A. thaliana* Col‐0 plants (two hydroponic vessels) per treatment at Zeitgeber Time ZT 7 (time after lights on), pooled and immediately frozen in liquid nitrogen and stored at −80°C until further processing. Tissues were homogenized in liquid nitrogen using a mortar and pestle. Total RNA was extracted from aliquots of 50–100 mg of ground tissue per sample using TRIzol Reagent (Fisher Scientific, Schwerte, Germany). RNA quality and quantity were verified by Nanodrop spectrophotometry and using an Agilent 2100 Bioanalyzer. RNA‐seq libraries were constructed with the VAHTS Universal V8 RNA‐seq Library Prep Kit (Vazyme, Nanjing, China) for strand‐specific sequencing according to the manufacturer's instructions, followed by sequencing on the Illumina NovaSeq platform at Biomarker Technologies (BMK) GmbH (Münster, Germany). Adaptor sequences were removed, and low‐quality ends were trimmed from raw reads by Biomarker Technologies (BMK GmbH, Münster, Germany) according to their standard protocols.

The resulting clean reads (*ca*. 7 Gbp of paired‐end 150‐bp reads, 24.6–28.5 mio. read pairs, per sample) were mapped to the *A. thaliana* TAIR10.1 reference genome using STAR v2.7.3a (Dobin et al. 2013), and gene‐level read counts were obtained with featureCounts v2.0.0 (Liao et al. 2014) using exon features (‐t exon), strand‐specific counting (‐s 2), and paired‐end mode (‐p). Genes with counts ≥ 10 in at least three samples were retained for differential expression analysis, which was performed in R v4.5.1 using DESeq2 v1.48.2 (Love et al. 2014) with a full interaction design ~ batch + Fe * pH. Genes with |Log_2_(fold change)| > Log_2_(1.5) ≈ 0.585 and adjusted *p* value < 0.05 were considered significantly differentially expressed. Significantly differentially expressed genes (DEGs) between any two cultivation conditions (excluding the comparison L Fe pH 5.5 vs. W Fe pH 7.5) were clustered using the soft clustering method Mfuzz (Kumar and Futschik 2007). For each cluster, gene ontology (GO) and Kyoto Encyclopedia of Genes and Genomes (KEGG) pathway enrichment analyses were performed using clusterProfiler v4.16.0 (Yu et al. 2012) with Benjamini–Hochberg adjusted *p* values (FDR) of < 0.05 (GO) and 0.1 (KEGG). Cluster‐specific expression patterns were visualized using ClusterGVis‐app GitHub v0.1 (Zhang et al. 2026), which internally applies ComplexHeatmap v2.20.0 (Gu et al. 2016). Sequence data from this article can be found at ENA, EMBL‐EBI under accession number PRJEB111645.

### 4.3 Quantitative RT‐PCR

Total RNA used for RT‐qPCR analysis was an aliquot of the solution used also for RNA‐seq. DNase treatment was performed using the TURBO DNA‐free Kit (Thermo Fisher Scientific, Waltham, MA, USA) following the manufacturer's instructions. First‐strand cDNA was synthesized from 1 µg of DNase‐treated RNA using the RevertAid First Strand cDNA Synthesis Kit with the oligo (dT)_18_ primers provided in the kit (Thermo Fisher Scientific, Waltham, MA, USA). Quantitative PCR was carried out on a LightCycler 480 system (Roche, Basel, Switzerland) in 10‐µL reaction volumes containing diluted cDNA and GoTaq qPCR Master Mix (Promega, Madison, WI, USA). Each reaction contained 8 ng of cDNA and gene‐specific primers (0.25 µM each). The thermal cycling programme included an initial incubation at 50°C for 2 min and 95°C for 10 min, followed by 40 cycles of 95°C for 15 s and 60°C for 1 min. A final dissociation step was included for melting‐curve analysis. PCR amplification efficiencies were calculated using LinRegPCR software version 2016.0 (Ruijter et al. 2009), and used to calculate the transcript level as TL = *E* ^−C*t*^, with C*t* as the cycle threshold. Relative transcript levels (RTLs) were calculated by dividing the transcript level of the gene of interest by the geometric mean of transcript levels of the two reference genes, *UBQ10* and *TIP41‐like*. Primer sequences are listed in Table S3.

### 4.4 Root Elongation, Biomass, Chlorophyll Content and Multi‐Element Analysis

We carried out dedicated experiments comprising one replicate hydroponic vessel containing three to four replicate plants of the same genotype per treatment, with two independent experiments for *A. halleri* and one independent experiment for *A. thaliana* for reference, respectively. Plants were photographed before and after treatments by carefully transferring them from hydroponic culture into square 250‐mm polystyrene Petri dishes (Greiner Bio‐One GmbH, Frickenhausen, Germany) containing ultrapure water. Images were acquired using a Nikon Digital SLR camera equipped with an AF‐S DX Zoom NIKKOR 18–70 mm 1:3.5–4.5 G ED‐IF objective at a distance of 50 mm. Root system length was quantified in ImageJ (Schneider et al. 2012) by tracing a segmented line along the three longest roots of each plant.

For the quantification of leaf chlorophyll contents, shoot tissues were harvested into 50‐mL Falcon tubes and flash‐frozen in liquid nitrogen immediately after harvest. The frozen shoots were ground into a fine powder in liquid nitrogen using a mortar and pestle. Approximately 10–30 mg of homogenized shoot tissues were transferred into a 2‐mL polypropylene tube and kept in liquid nitrogen. After adding 1.5 mL of 100% (v/v) methanol (Merck), the samples were covered with aluminium foil and incubated at 70°C for 15 min in a thermomixer, with mixing at 850 rpm for 10 s every 5 min. After cooling on ice for 5 min, the methanol extracts were cleared by centrifugation at 14 000 rpm and 4°C for 1 min. Absorbance was measured in three technical replicates at 652 and 665 nm using a Synergy HTX Multi‐Mode Reader (Agilent, formerly BioTek) with microplate path length correction (Warren 2008), followed by the calculation of chlorophyll concentrations (Porra et al. 1989).

Fresh biomass of each shoot and root was quantified directly after harvest. To remove apoplastically bound metals, each root was incubated in 20 mL ice‐cold desorption solution I (2 mM CaSO_4_, 10 mM Na_2_EDTA, 1 mM MES; pH 5.7) with gentle shaking at 80 rpm on ice for 10 min. Roots were then transferred to fresh 50‐mL tubes containing 20 mL desorption solution II (0.3 mM bathophenanthroline disulphonate, 5.7 mM sodium dithionite) and incubated with gentle shaking at 80 rpm on ice for 10 min (Cailliatte et al. 2010). Root samples were subsequently rinsed in 100 mL ice‐cold ddH_2_O with gentle stirring for 1 min, blotted dry with tissue paper and placed into paper bags. Shoot and root tissues were oven‐dried at 60°C for 3 days and subsequently equilibrated in ambient air for ≥ 24 h. Approximately 20 mg of dried tissue powder per sample was transferred into PTFE MPV‐100 microwave vessels (MLS GmbH, Leutkirch, Germany) and digested with 3 mL 65% (w/w) HNO_3_ (analytical grade) using a Mars 5 microwave digestion system (CEM, Kamp‐Lintford, Germany). Multi‐element analysis was performed by ICP‐OES (iCAP 6500 Duo; Thermo Fisher Scientific, Waltham, MA, USA) as previously described (Stein et al. 2017). Instrument calibration and measurement accuracy were ensured by using a reagent blank, a series of multi‐element standard solutions, and digests of a certified reference material (INCT‐PVTL‐6, Virginia tobacco [*Nicotiana tabacum*] leaves; Institute of Nuclear Chemistry and Technology, Poland).

### 4.5 Root Hair Imaging and Measurements

Root segments of 20–30 mm from the root tip were excised from *A. halleri* and *A. thaliana* directly after harvest for imaging. The four to six longest roots were randomly selected from three clones of *A. halleri*, and for *A. thaliana*, four to six roots were selected from three to four plants. Images were acquired using a Carl Zeiss Axio Imager microscope and an AxioCam MR R3 camera. Root hair longitudinal density was determined by counting the total number of root hairs within a 0.5 mm segment starting from the onset of the differentiation zone near the root apex using ZEN 3.8 software.

### 4.6 Statistical Analyses

All statistical analyses were performed in R v4.5.1 or IBM SPSS Statistics v31 (Data S5). For datasets meeting the assumptions of normality and homogeneity of variance (assessed using residual plots or normality tests), analysis of variance (ANOVA) followed by Duncan's or Tukey's post hoc test (*p* < 0.05) was applied. Pairwise comparisons between specific groups were performed using two‐tailed Student's *t*‐tests in cases where differences were not detected by ANOVA due to high variance within other groups. Data were visualized as bar or line plots using GraphPad Prism 10. All scripts are available here: https://github.com/genyangyg/Iron_pH_Ath_transcriptomics.

## Supporting information


**Figure S1:** Supplementary transcriptome analyses and RT‐qPCR validation of results from RNA sequencing.
**Figure S2:** KEGG pathway enrichment of clusters of differentially abundant transcripts in response to low Fe, high pH, or their combination in *A. thaliana*.
**Figure S3:** Geographic and soil information for the sites of origin of the *A. halleri* and *A. thaliana* accessions used here.
**Figure S4:** Root growth and representative images for different accessions of *A. halleri* and *A. thaliana*.
**Figure S5:** Root growth and leaf chlorophyll content in accessions of *A. halleri* and *A. thaliana* exposed to low Fe and high pH in hydroponic media.
**Figure S6:** Shoot ionomes of *A. halleri* and *A. thaliana* exposed to low Fe and high pH in hydroponic media.
**Figure S7:** Root ionomes of *A. halleri* and *A. thaliana* exposed to low Fe and high pH in hydroponic media.
**Figure S8:** Integrative PCA biplots of shoot‐to‐root ionome ratios, shoot and root ionomes across all accessions of *A. halleri* and *A. thaliana* exposed to low Fe and high pH in hydroponic media.
**Figure S9:** Images of the root hair zones of *A. halleri* and *A. thaliana* exposed to low Fe and high pH in hydroponic media.
**Figure S10:** Fe and Mn concentrations in accessions of *A. halleri* and *A. thaliana* exposed to low Fe and high pH in hydroponic media.
**Figure S11:** Relationships between root hair density, root Mo concentrations and root Mn concentrations in *A. halleri* and *A. thaliana* exposed to low Fe and high pH in hydroponic media.
**Figure S12:** Effect of pH buffering of hydroponic solutions on Mn and Fe homeostasis in *A. thaliana* exposed to low Fe and high pH.
**Table S1:** Linear regression models for the field data (Stein et al. 2017).
**Table S2:** Linear regression models for experimental data from this study.
**Table S3:** Oligonucleotides used in the present study.

**Data S1:** Top principal component (PC1–PC3) genes in roots of *Arabidopsis thaliana* (Col‐0) grown under conditions with low Fe and/or high pH for 5 days.

**Data S2:** Universal gene expression profiles in roots of *Arabidopsis thaliana* (Col‐0) grown under conditions with low Fe and/or high pH for 5 days.

**Data S3:** Significantly enriched GO terms of 8 clusters.

**Data S4:** Significantly enriched KEGG terms of 8 clusters.

**Data S5:** Raw data and statistics.

## Data Availability

The data that support the findings of this study are available in the Supporting Information of this article, and further data have been uploaded to public repositories and will be publicly available with the publication of this article.
