# Supplementary material for: Cross‐Species Plasticity and Divergence Under Alkaline Soil Signature Conditions Differentiating PYE From FIT Target Transcripts in the Arabidopsis thaliana Ferrome
Source: Plant Cell Environ. 2026 Jun 4;49(9):6719–36. doi: 10.1111/pce.70645 (PMC13436447; doi:10.1111/pce.70645)
Supplement: Supplementary file 1 [file PCE-49-6719-s005.pdf]

Cross-species plasticity and divergence under alkaline soil signature conditions differentiating PYE from FIT target transcripts in the *Arabidopsis thaliana* ferrome

Gen Yang[1], Hans-Simon Metze[1] and Ute Krämer[1]

[1]Molecular Genetics and Physiology of Plants, Faculty of Biology and Biotechnology, Ruhr University Bochum, D-44801 Bochum, Germany

Supplementary Information

In this .pdf file:

Fig. S1 Supplementary transcriptome analyses and RT-qPCR validation of results from RNA sequencing

Fig. S2 KEGG pathway enrichment of clusters of differentially abundant transcripts in response to low Fe, high pH, or their combination in *A. thaliana*

Fig. S3 Geographic and soil information for the sites of origin of the *A. halleri* and *A. thaliana* accessions used here

Fig. S4 Root growth and representative images for different accessions of *A. halleri* and *A. thaliana*

Fig. S5 Root growth and leaf chlorophyll content in accessions of *A. halleri* and *A. thaliana* exposed to low Fe and high pH in hydroponic media

Fig. S6 Shoot ionomes of *A. halleri* and *A. thaliana* exposed to low Fe and high pH in hydroponic media

Fig. S7 Root ionomes of *A. halleri* and *A. thaliana* exposed to low Fe and high pH in hydroponic media

Fig. S8 Integrative PCA biplots of shoot-to-root ionome ratios, shoot and root ionomes across all accessions of *A. halleri* and *A. thaliana* exposed to low Fe and high pH in hydroponic media

Fig. S9 Images of the root hair zones of *A. halleri* and *A. thaliana* exposed to low Fe and high pH in hydroponic media

Fig. S10 Fe and Mn concentrations in accessions of *A. halleri* and *A. thaliana* exposed to low Fe and high pH in hydroponic media

Fig. S11 Relationships between root hair density, root Mo concentrations and root Mn concentrations in *A. halleri* and *A. thaliana* exposed to low Fe and high pH in hydroponic media

Fig. S12 Effect of pH buffering of hydroponic solutions on Mn and Fe homeostasis in *A. thaliana* exposed to low Fe and high pH

Table S1 Linear regression models for the field data (Stein et al., 2017)

Table S2 Linear regression models for experimental data from this study

Table S3 Oligonucleotides used in the present study

Provided as separate files:

Suppl. Data S1 Top principal component (PC1–PC3) genes in roots of *Arabidopsis thaliana* (Col-0) grown under conditions with low Fe and/or high pH for 5 days

Suppl. Data S2 Universal gene expression profiles in roots of *Arabidopsis thaliana* (Col-0) grown under conditions with low Fe and/or high pH for 5 days

Suppl. Data S3 Significantly enriched GO terms of 8 clusters

Suppl. Data S4 Significantly enriched KEGG terms of 8 clusters

Suppl. Data S5 Raw data and statistics

Figure S1, Yang *et al.*

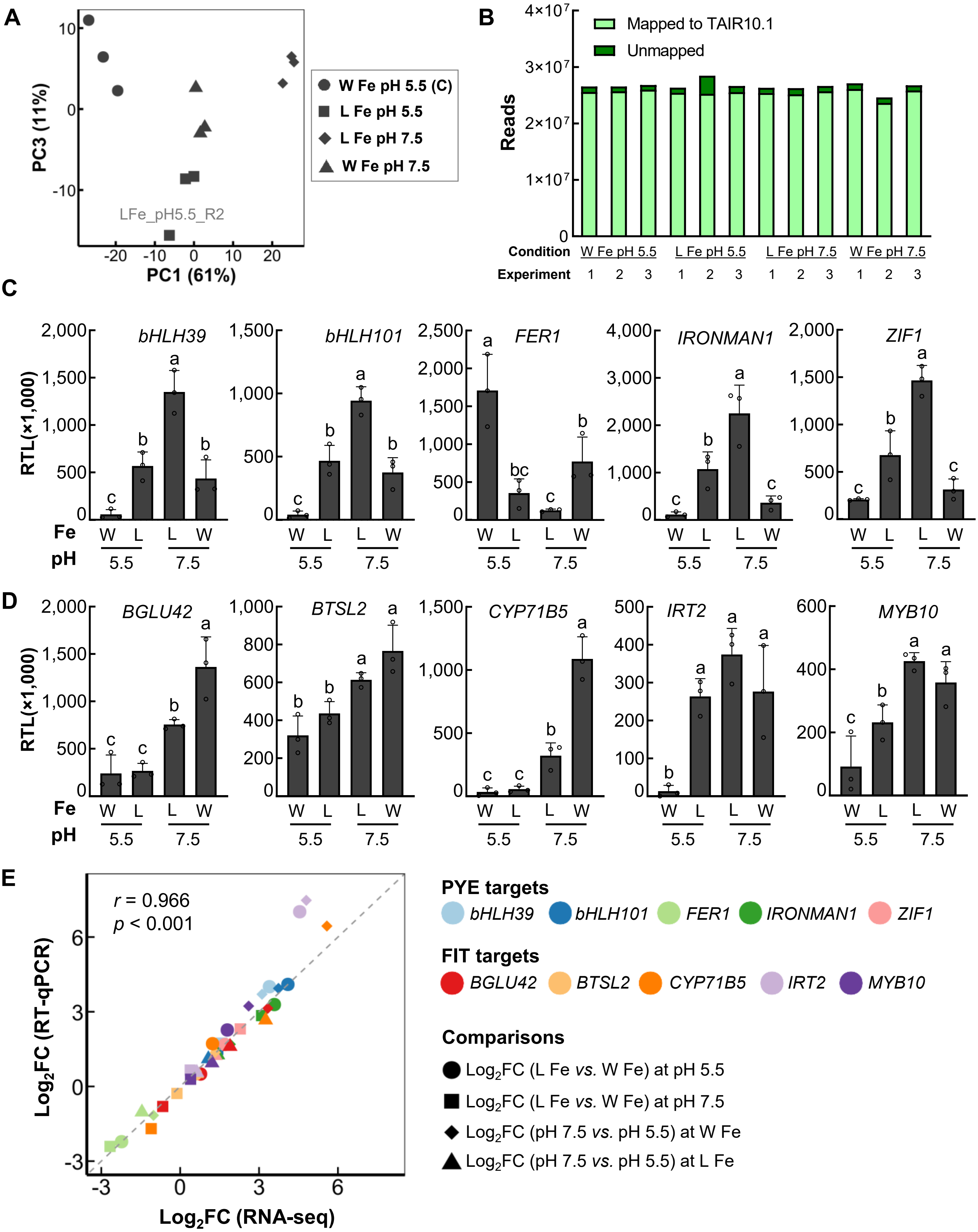

Fig. S1 Supplementary transcriptome analyses and RT-qPCR validation of results from RNA sequencing. (A) Principal Component Analysis (PCA) showing PC1 and PC3 of root transcriptomes after 5 d of exposure to a single low-Fe treatment (L Fe pH 5.5), a single high-pH treatment (with iron, W Fe pH 7.5), and their combination (L Fe pH 7.5), alongside a control treatment (W Fe pH 5.5, based on the top 500 most variable transcripts after variance-stabilizing transformation; see Fig. 1A and Suppl. Data S1). **(B)** Read mapping in all samples after RNA sequencing. **(C-D)** Relative transcript levels of PYE target genes (C) and FIT target genes (D) quantified using RT-qPCR. Transcript levels were normalized to the reference genes *UBQ10* and *TIP41-like*. Bars represent arithmetic mean ± SD (n = 3 independent experiments, with one pool of root tissues from six replicate *A. thaliana* (Col-0) plants per treatment in each experiment). RT-qPCR was performed with three technical replicates per biological sample, using cDNA synthesized from replicate aliquots of the RNA solutions used for RNA-seq. Distinct characters denote statistically significant differences ($p < 0.05$) according to ANOVA (Duncan's test) **(E)** Correlation between RT-qPCR- and RNA-seq-derived Log_2(fold change). Pearson's correlation coefficient was used to assess the correlation between the two methods. The dashed line indicates a 1:1 relationship.

Figure S2, Yang et al.

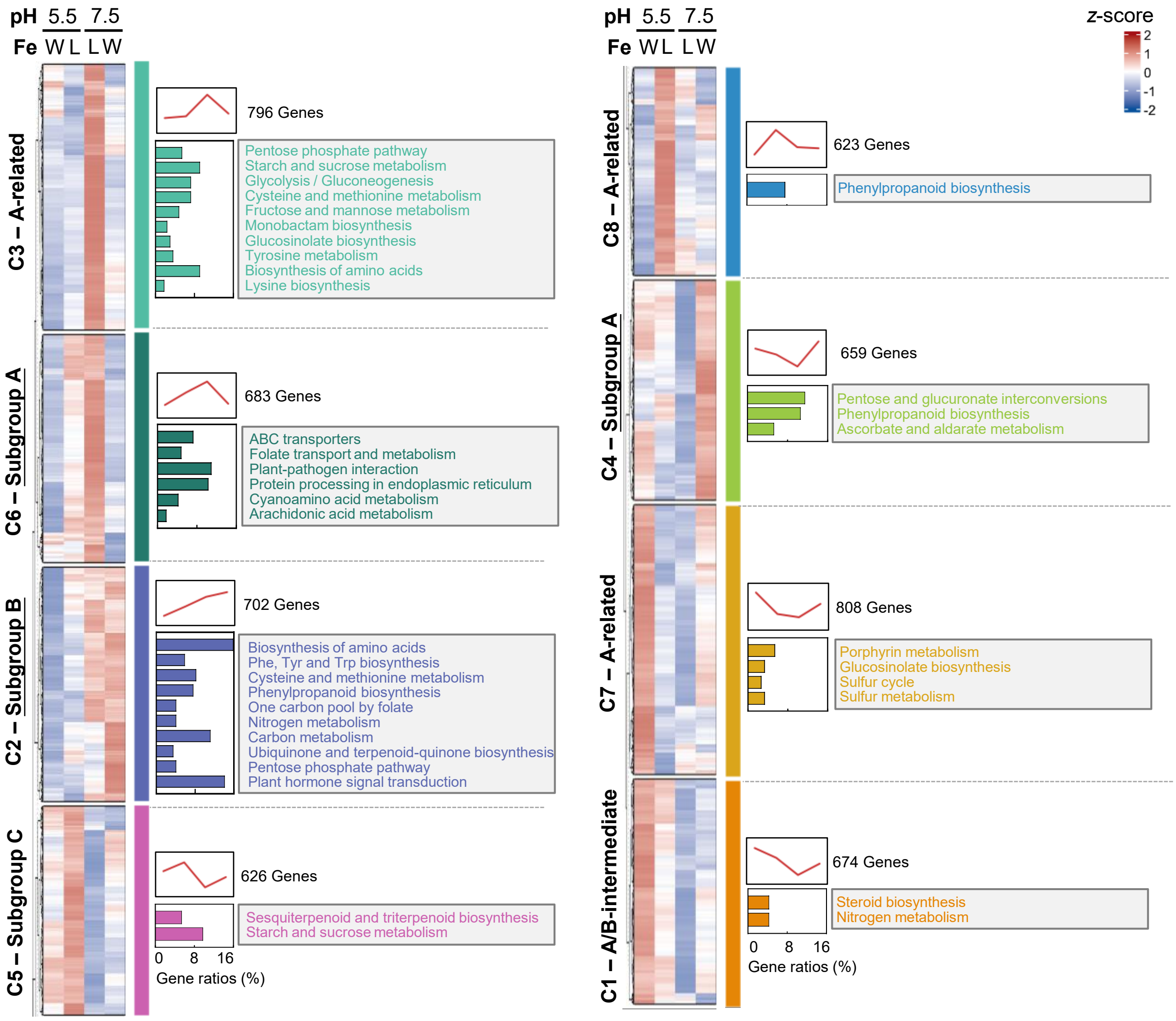

Fig. S2 KEGG pathway enrichment of clusters of differentially abundant transcripts in response to low Fe, high pH, or their combination in *A. thaliana*. Heatmaps generated by Mfuzz soft clustering of 5,571 differentially abundant transcripts (left), with the total number of transcripts, a line plot illustrating the representative expression pattern, as well as the top significantly enriched KEGG pathways listed alongside bar plots showing gene ratios (right) (see Fig. 1D, see Suppl. Data S4). Differential abundance: $|Log_2(\text{fold change})| > Log_2(1.5) \approx 0.585$ and adjusted p-value < 0.05 ($n = 3$ independent experiments, with one pool of root tissues from six replicate *A. thaliana* (Col-0) plants per treatment per experiment).

Figure S3, Yang et al.

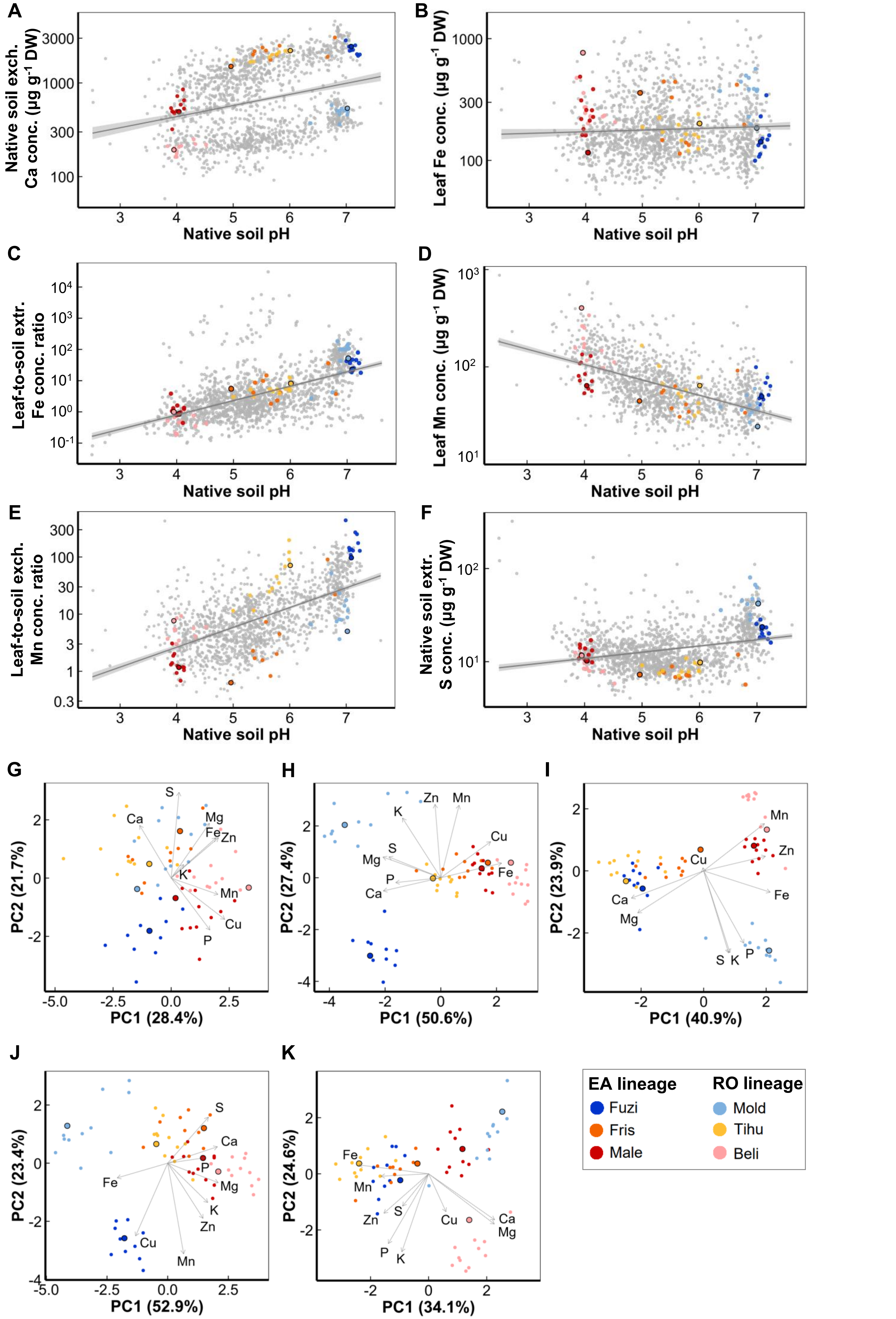

Fig. S3 Geographic and soil information for the sites of origin of the *A. halleri* and *A. thaliana* accessions used here. (**A-F**) Linear correlations with soil pH of native soil exchangeable Ca concentrations (A), leaf Fe concentrations (B), the ratios of leaf Fe concentration to Fe concentration in the soil fraction extractable by 0.1 M HCl (C), leaf Mn concentrations (D), the ratios of leaf Mn concentration to Mn concentration in the soil fraction exchangeable in 0.01 M $BaCl_2$ (E), soil extractable S concentration (F), at the field sites of origin of *A. halleri*. (**G-K**) PCA biplots of leaf ionomes (G), soil extractable (H) and soil exchangeable (I) concentrations, as well as of ratios of leaf:soil extractable (J) and leaf:soil exchangeable (K) element concentration ratios. PCA was performed on *z*-scores of $Log_{10}(x)$-transformed element concentrations. Leaf-to-soil ratios for extractable and exchangeable element concentrations in soil were calculated based on Log_{10}[(leaf ion conc.) / (soil extractable/exchangeable ion conc.)]. Labeled arrows indicate the contributions of individual variables to the loadings of PC1 and PC2 ($n \sim 12$ replicate plant individuals per site; data from Stein et al., 2017). Each data point (A-K) represents one *A. halleri* individual ($n = 1{,}466$ plant individuals collected at non-metalliferous sites in the field; Stein et al., 2017). Linear regression models are given in Table S2 (A-F). DW, dry mass.

Figure S4, Yang et al.

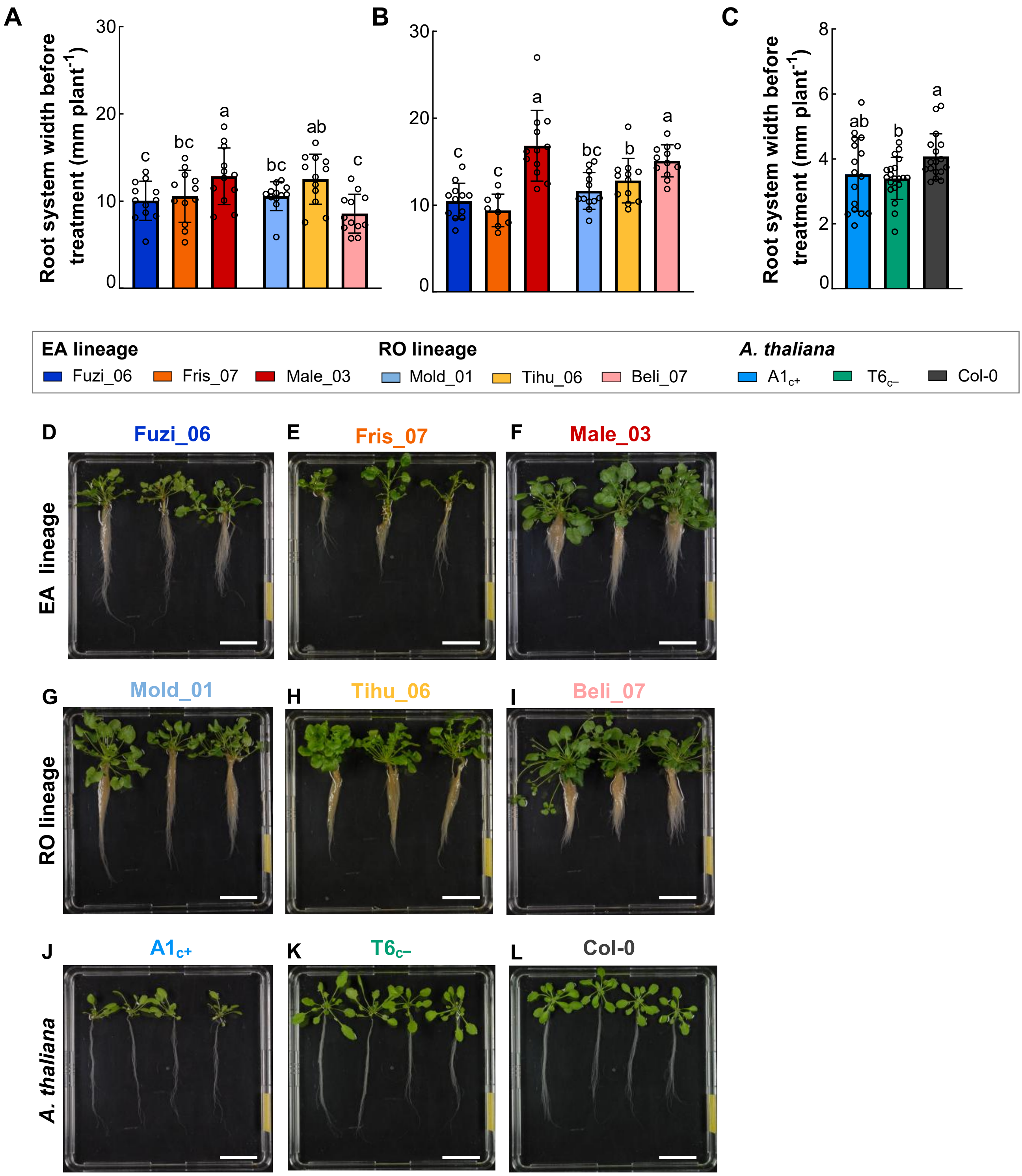

Fig. S4 Root growth and representative images for different accessions of *A. halleri* and *A. thaliana*. (A-C) Maximum width of the root systems of four-week-old *A. halleri* (A, B; each corresponding to an independent experiment) and five-week-old *A. thaliana* (C) plants upon pre-cultivation in control hydroponic solutions prior to treatment. **(D-L)** Representative images of the accessions from the EA (D-F) and the RO (G-I) lineages of *A. halleri* (from the same experiment as shown in B), and of *A. thaliana* (J-L) accessions prior to treatment. Bars show arithmetic mean ± SD of n = 8 to 12 replicate clones (A, B), and n = 16 to 20 replicate plants (C). Distinct letters denote statistically significant differences ($p < 0.05$) according to ANOVA (Duncan's test), analyses across groups (separated by vertical dashed grey lines), as follows: all *A. halleri* (A, B), all *A. thaliana* (C). Scale bars: 5 cm.

Figure S5, Yang et al.

A

Fe	pH	*A. halleri*_Experiment 1					
		EA lineage			RO lineage		
		Fuzi_06	Fris_07	Male_03	Mold_01	Tihu_06	Beli_07
W	5.5	6.39^{a}	3.59^{b}	4.16^{a}	7.04^{a}	3.25^{a}	7.04^{a}
L	5.5	2.54^{b}	4.81^{a}	2.68^{b}	5.67^{a}	3.99^{a}	2.35^{b}
L	7.5	3.70^{ab}	3.15^{b}	1.50^{b}	1.92^{b}	1.56^{a}	1.86^{b}
W	7.5	0.73^{b}	3.20^{b}	0.93^{b}	0.40^{b}	1.73^{a}	2.71^{b}

B

Fe	pH	*A. thaliana*		
		A1$_{c+}$	T6$_{c-}$	Col-0
W	5.5	2.41^{a}	4.63^{a}	3.44^{a}
L	5.5	1.73^{b}	2.74^{b}	2.15^{b}
L	7.5	0.30^{c}	1.05^{c}	0.63^{c}
W	7.5	0.43^{c}	0.92^{c}	0.45^{c}
W	+Bic	0.28^{c}	0.77^{c}	0.28^{c}

C

Fe	pH	*A. halleri*_Experiment 2					
		EA lineage			RO lineage		
		Fuzi_06	Fris_07	Male_03	Mold_01	Tihu_06	Beli_07
W	5.5	7.60^{a}	3.40^{a}	1.97^{a}	4.57^{a}	5.77^{a}	5.07^{a}
L	5.5	6.77^{ab}	3.01^{a}	0.63^{b}	2.60^{ab}	1.29^{c}	2.06^{c}
L	7.5	4.51^{bc}	1.69^{b}	0.54^{b}	2.33^{b}	3.52^{b}	3.63^{b}
W	7.5	2.18^{c}	2.79^{ab}	0.87^{ab}	0.75^{b}	1.60^{c}	4.88^{a}

Root elongation rate (z-score): 2.5, 0, -2.5

D

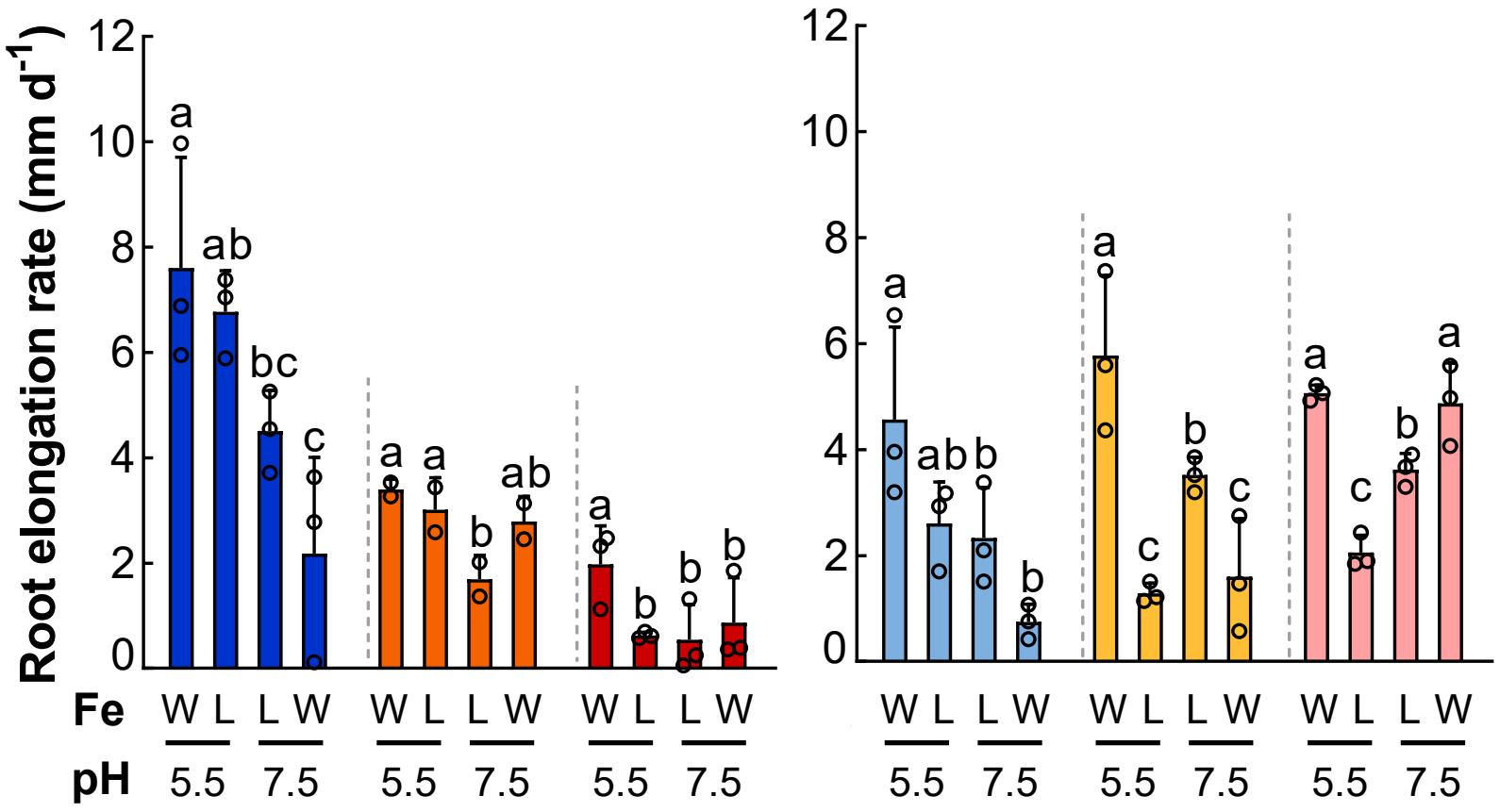

E

Fe	pH	*A. halleri*					
		EA lineage			RO lineage		
		Fuzi_06	Fris_07	Male_03	Mold_01	Tihu_06	Beli_07
W	5.5	1.25^{a}	0.89^{ab}	0.68^{b}	1.12^{ab}	0.58^{b}	1.08^{ab}
L	5.5	1.03^{ab}	0.83^{ab}	0.77^{ab}	1.00^{ab}	0.60^{b}	1.27^{a}
L	7.5	0.88^{ab}	0.88^{ab}	0.68^{b}	1.03^{ab}	0.58^{b}	1.25^{a}
W	7.5	1.00^{ab}	1.02^{ab}	0.76^{ab}	1.12^{ab}	0.72^{ab}	1.08^{ab}

F

Fe	pH	*A. thaliana*		
		A1$_{c+}$	T6$_{c-}$	Col-0
W	5.5	0.91^{b}	0.81^{cde}	1.02^{a}
L	5.5	0.63^{gh}	0.55^{h}	0.72^{ef}
L	7.5	0.67^{fg}	0.58^{h}	0.72^{ef}
W	7.5	0.75^{def}	0.69^{fg}	0.82^{cd}
W	+Bic	0.80^{de}	0.62^{gh}	0.89^{bc}

Chlorophyll content (z-score): 2.5, 0, -2.5

Fig. S5 Root growth and leaf chlorophyll content in accessions of *A. halleri* and *A. thaliana* exposed to low Fe and high pH in hydroponic media. (**A-C**) Root elongation rates. Colors represent z-scores calculated separately per experiment for *A. halleri* (A, C) and *A. thaliana* (B). Numbers are arithmetic means of root elongation rate (mm d^{-1}) (n = 3 replicate clones for *A. halleri* and replicate plants for *A. thaliana*; with data based on the mean elongation rate of the three longest root axes per clone or plant). Data in (A) and (B) are shown here again from Fig. 3). (**D**) Root elongation rate of the longest root per plant of *A. halleri*. Bars show arithmetic mean ± SD of n = 3 replicate clones (D) for a second independent experiment (see Fig. 3D. The same data are also shown in (C). (**E**, **F**) Leaf chlorophyll content (data shown again from Fig. 3F and G). Colors represent z-scores calculated separately for *A. halleri* (E) and *A. thaliana* (F). Numbers are arithmetic means of chlorophyll content (mg g^{-1} fresh biomass; n = 3 replicate clones for *A. halleri* from one experiment shown also in Fig. 3F; and n = 3 independent experiments, based on one arithmetic mean of 3 replicate plants per experiment). Before the calculation of z-scores, values were first normalized to the control (W Fe, pH 5.5) for each accession (A-C, E, F). Distinct letters denote statistically significant differences ($p < 0.05$) according to ANOVA (Duncan's test). W Fe, control Fe (10 µM FeHBED); L Fe, low Fe (0.1 µM FeHBED); EA, Eastern Alps; RO, Romania; c^{+}, from calcareous soil; c^{-}, from non-calcareous soil; +Bic, supplemented with 10 mM bicarbonate ($NaHCO_3$).

Figure S6, Yang et al.

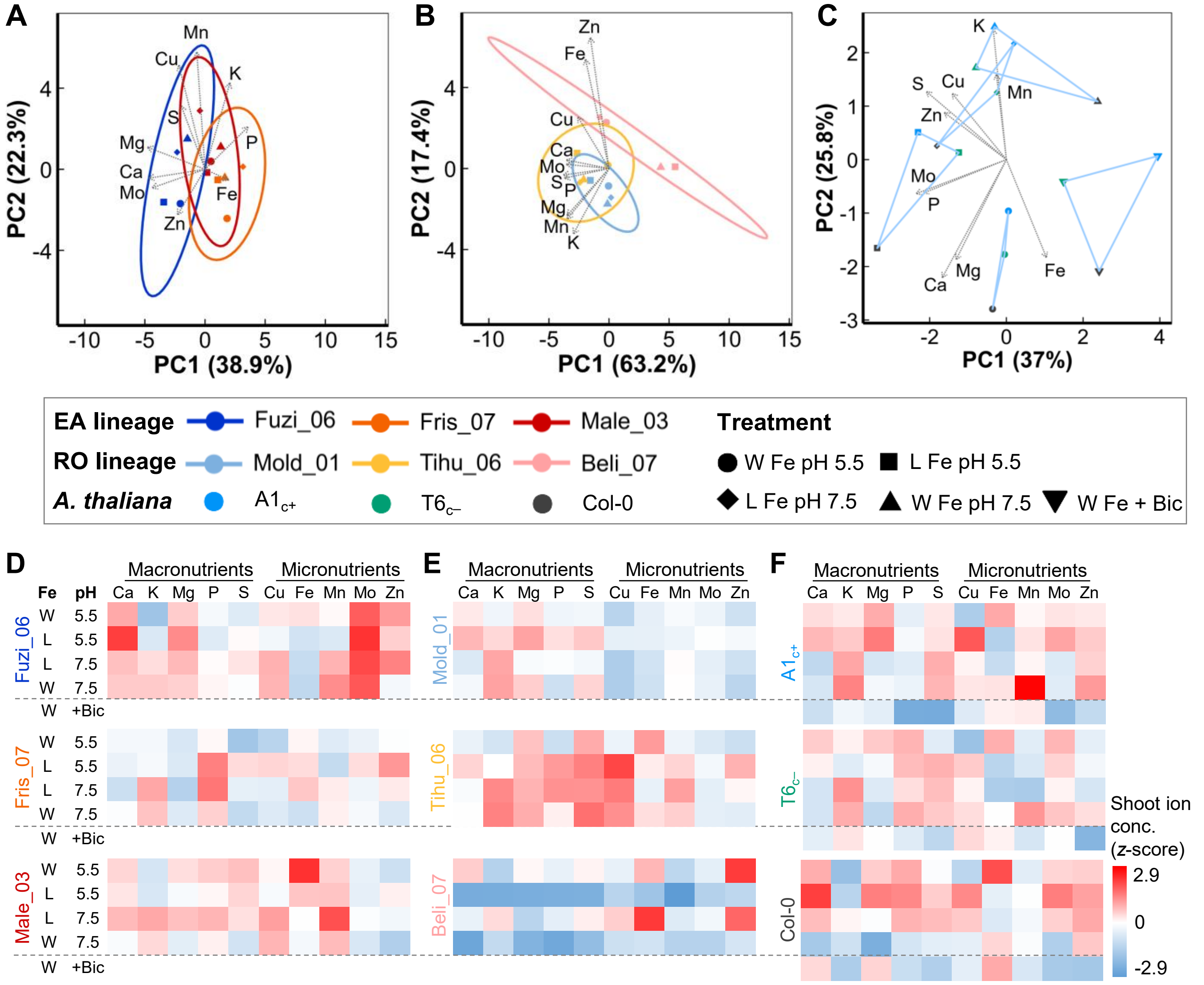

Fig. S6 Shoot ionomes of *A. halleri* and *A. thaliana* exposed to low Fe and high pH in hydroponic media. (**A**-**C**) Principal Component Analysis (PCA) biplots of shoot concentrations of each element in lineages EA (A) and RO (B) of *A. halleri* and in *A. thaliana* (C). Ellipses show 95% confidence limits around group centroids. Lines forming light blue triangles connect data points from different genotypes for a common treatment (shown for visual guidance). Arrows indicate the loadings of elements contributing to PC1 and PC2. (**D**-**F**) Heatmaps of elemental concentrations in shoots in the EA (D) and RO (E) lineages of *A. halleri* and in *A. thaliana* (F). PCA and heatmaps are based on *z*-scores calculated separately for each species from Log_{10}-transformed shoot elemental concentrations. Shown are arithmetic means (*n* = 3 replicate clones for *A. halleri*; and *n* = 3 independent experiments for *A. thaliana*, each represented by the mean of three replicate plants). W Fe, control Fe (10 µM FeHBED); L Fe, low Fe (0.1 µM FeHBED); EA, Eastern Alps; RO, Romania; c^+, from calcareous soil; c^-, from non-calcareous soil; +Bic, supplemented with 10 mM bicarbonate ($NaHCO_3$).

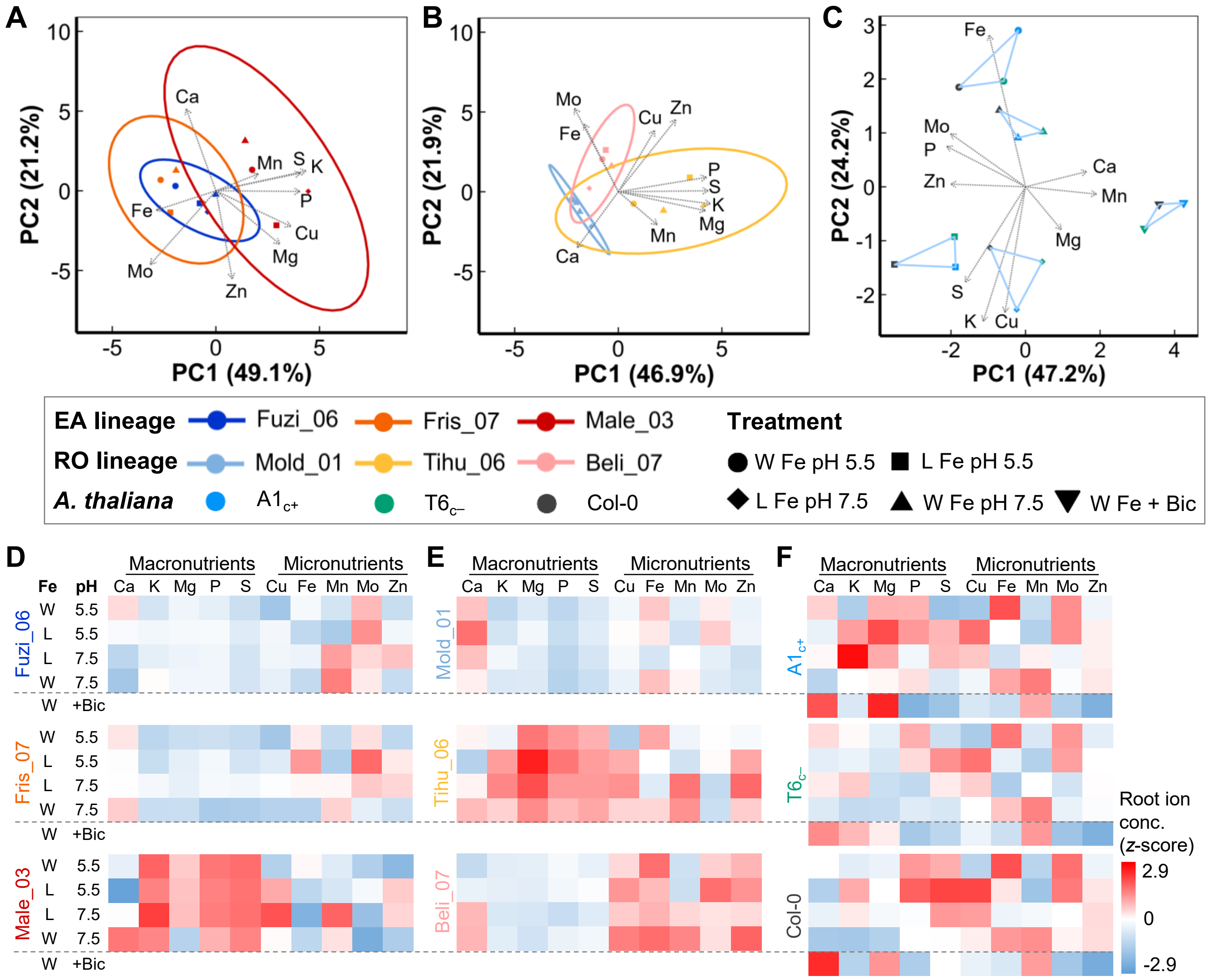

Fig. S7 Root ionomes of *A. halleri* and *A. thaliana* exposed to low Fe and high pH in hydroponic media. (**A-C**) Principal Component Analysis (PCA) biplots of root concentrations of each element in lineages EA (A) and RO (B) of *A. halleri* and in *A. thaliana* (C). Ellipses show 95% confidence limits around group centroids. Lines forming light blue triangles connect data points from different genotypes for a common treatment (shown for visual guidance). Arrows indicate the loadings of elements contributing to PC1 and PC2. (**D-F**) Heatmaps of elemental concentration in roots in the EA (D) and RO (E) lineages of *A. halleri* and in *A. thaliana* (F). PCA and heatmaps are based on *z*-scores calculated separately for each species from Log_{10}-transformed root elemental concentrations. Shown are arithmetic means (*n* = 3 replicate clones for *A. halleri*; and *n* = 3 independent experiments for *A. thaliana*, each represented by the mean of three replicate plants). W Fe, control Fe (10 µM FeHBED); L Fe, low Fe (0.1 µM FeHBED); EA, Eastern Alps; RO, Romania; c⁺, from calcareous soil; c⁻, from non-calcareous soil; +Bic, supplemented with 10 mM bicarbonate ($NaHCO_3$).

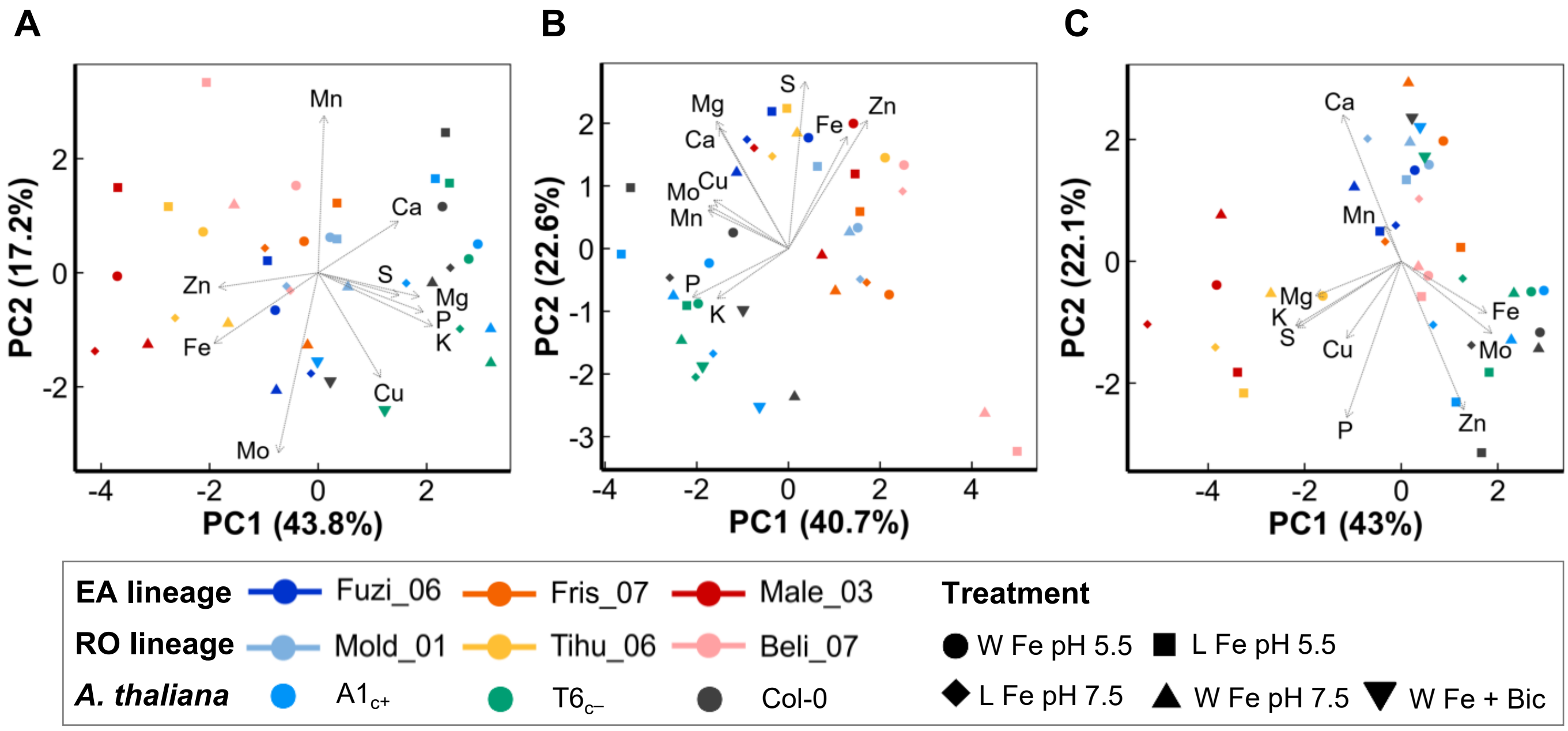

Fig. S8 Integrative PCA biplots of shoot-to-root ionome ratios, shoot and root ionomes across all accessions of *A. halleri* and *A. thaliana* exposed to low Fe and high pH in hydroponic media. (**A-C**) Principal Component Analysis (PCA) of shoot-to-root elemental concentration ratios (A) reflecting root-to-shoot partitioning of nutrient elements, shoot elemental concentrations (B), and root elemental concentrations (C) in *A. halleri* and *A. thaliana*. PCA was performed using mean *z*-scores of $\log_{10}$-transformed elemental concentration ratios (Log_{10}[(shoot ion conc.) / (root ion conc.]), and of Log_{10}-transformed shoot or root elemental concentrations, based on mean values from three (*A. halleri*) or nine replicates (*A. thaliana*) per accession × treatment combination (*n* = 3 replicate clones for *A. halleri*; and *n* = 3 independent experiments for *A. thaliana*, each represented by the mean of three replicate plants). Arrows indicate the loadings of elements contributing to PC1 and PC2. W Fe, control Fe (10 µM FeHBED); L Fe, low Fe (0.1 µM FeHBED); EA, Eastern Alps; RO, Romania; c^+, from calcareous soil; c^-, from non-calcareous soil; +Bic, supplemented with 10 mM bicarbonate ($NaHCO_3$).

Figure S9, Yang et al.

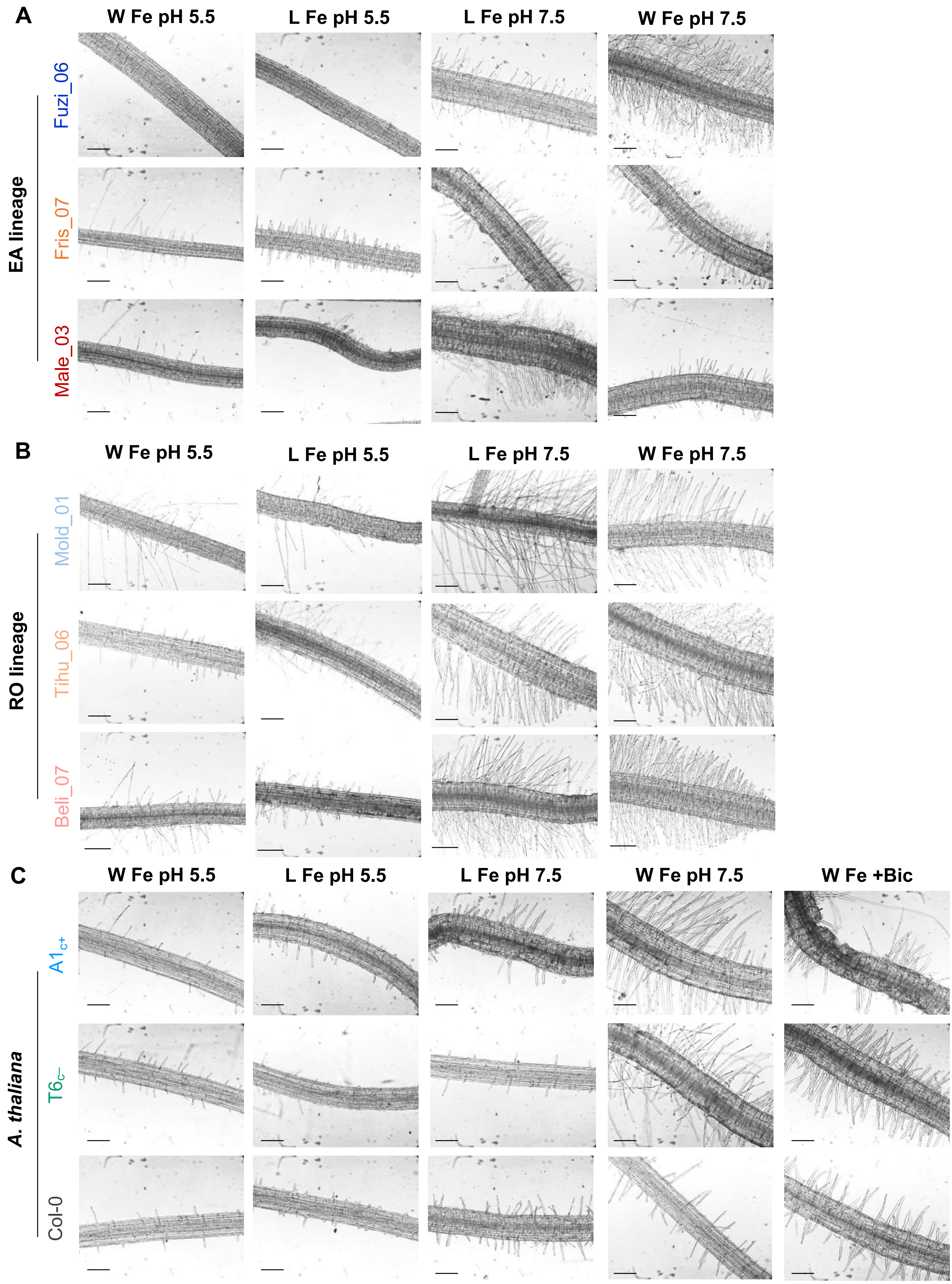

Fig. S9 Images of the root hair zones of *A. halleri* and *A. thaliana* exposed to low Fe and high pH in hydroponic media. (A-C) Representative bright-field images of the early differentiation zone of roots of the EA (A) and RO (B) lineages of *A. halleri*, and *A. thaliana* (C). For all samples, root segments were excised approximately 2 to 3 cm above the root tip, using a total of 4 to 6 of the longest roots from three replicate clones of *A. halleri*, or from three to four individual plants of *A. thaliana*. Scale bar: 100 µm. W Fe, control Fe (10 µM FeHBED); L Fe, low Fe (0.1 µM FeHBED); EA, Eastern Alps; RO, Romania; c^+, from calcareous soil; c^-, from non-calcareous soil; +Bic, supplemented with 10 mM bicarbonate ($NaHCO_3$).

Figure S10, Yang et al.

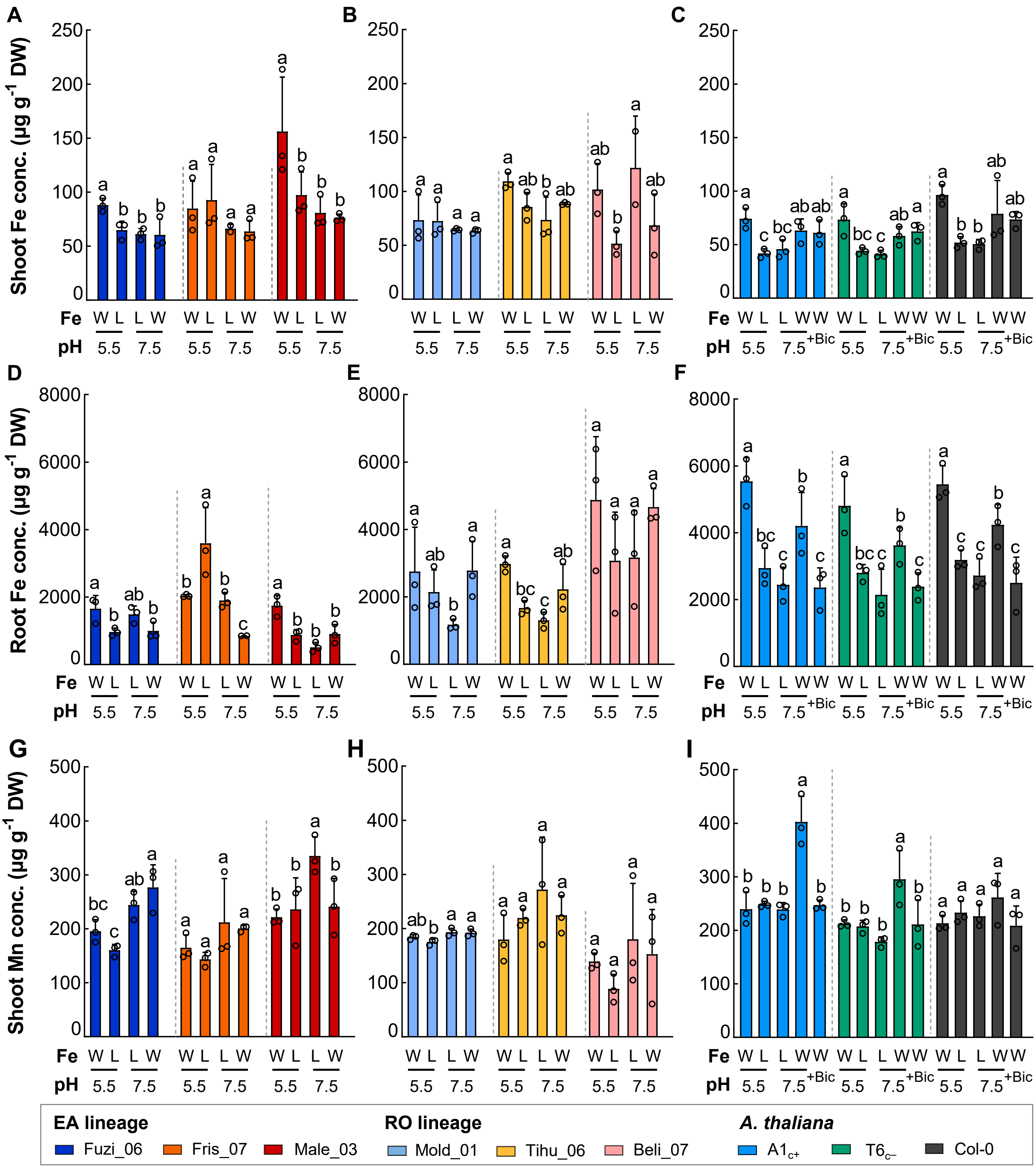

Fig. S10 Fe and Mn concentrations in accessions of *A. halleri* and *A. thaliana* exposed to low Fe and high pH in hydroponic media. (**A-I**) Fe concentrations in leaves (A-C) and roots (D-F), and Mn concentrations in leaves (G-I) of the EA (A, D, G) and RO (B, E, H) lineages of *A. halleri*, and in *A. thaliana* (C, F, I). Bar graphs show mean ± SD (*n* = 3 replicate clones for *A. halleri*; and *n* = 3 independent experiments for *A. thaliana*, each represented by the mean of three replicate plants). Distinct letters denote statistically significant differences ($p < 0.05$) according to ANOVA (Duncan's test). W Fe, control Fe (10 µM FeHBED); L Fe, low Fe (0.1 µM FeHBED); EA, Eastern Alps; RO, Romania; c$^+$, from calcareous soil; c$^-$, from non-calcareous soil; +Bic, supplemented with 10 mM bicarbonate ($NaHCO_3$).

Figure S11, Yang et al.

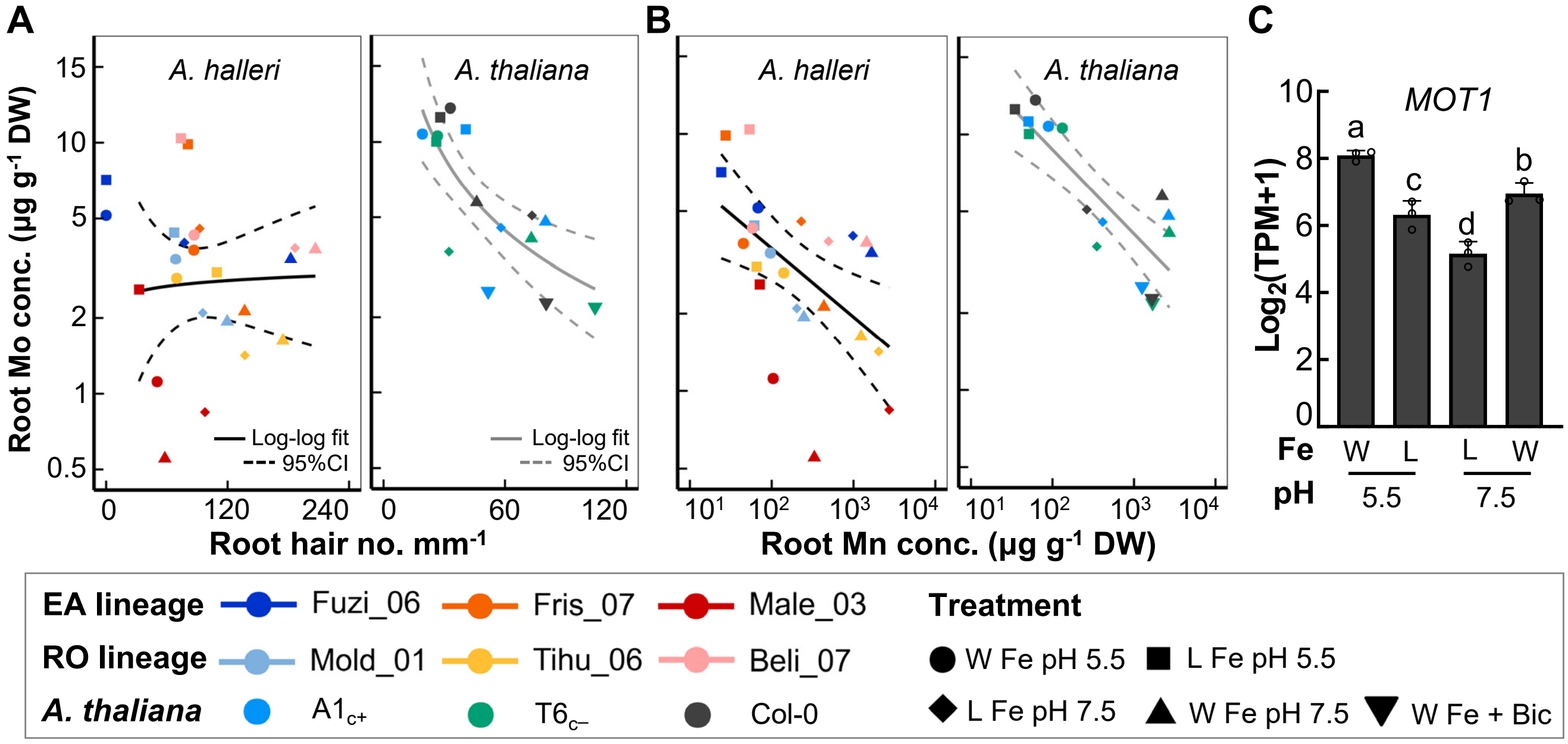

Fig. S11 Relationships between root hair density, root Mo concentrations and root Mn concentrations in *A. halleri* and *A. thaliana* exposed to low Fe and high pH in hydroponic media. (**A**, **B**) Scatterplots showing root Mo concentrations as a function of root hair number (A) and as a function of root Mn concentrations (B) across accessions of *A. halleri* (left) and *A. thaliana* (right). Regressions (solid black lines, fit) and 95% confidence interval (dashed lines, CI) were fitted on Log_{10}-transformed data (mean values) after excluding samples with zero root hairs (see Fig. 5), and subsequently back-transformed for plotting (all data shown; for concentrations, *n* = 3 replicate clones for *A. halleri* and independent experiments for *A. thaliana*, each represented by the mean of three replicate plants; for root hair density, *n* = 4 to 6 longest roots randomly selected among three replicate clones of *A. halleri*, and *n* = 3 to 4 replicate plants of *A. thaliana*). (**C**) Transcript levels of the molybdenum transporter *MOT1* in *A. thaliana* roots (Data S2). Bar plot shows arithmetic mean ± SD (*n* = 3 pools of six plants; with each pool from one independent experiment). Distinct characters denote statistically significant differences ($p < 0.05$) according to ANOVA (Duncan's test; C). W Fe, control Fe (10 µM FeHBED); L Fe, low Fe (0.1 µM FeHBED); EA, Eastern Alps; RO, Romania; c^+, from calcareous soil; c^-, from non-calcareous soil; +Bic, bicarbonate. TPM, transcripts per million.

Figure S12, Yang et al.

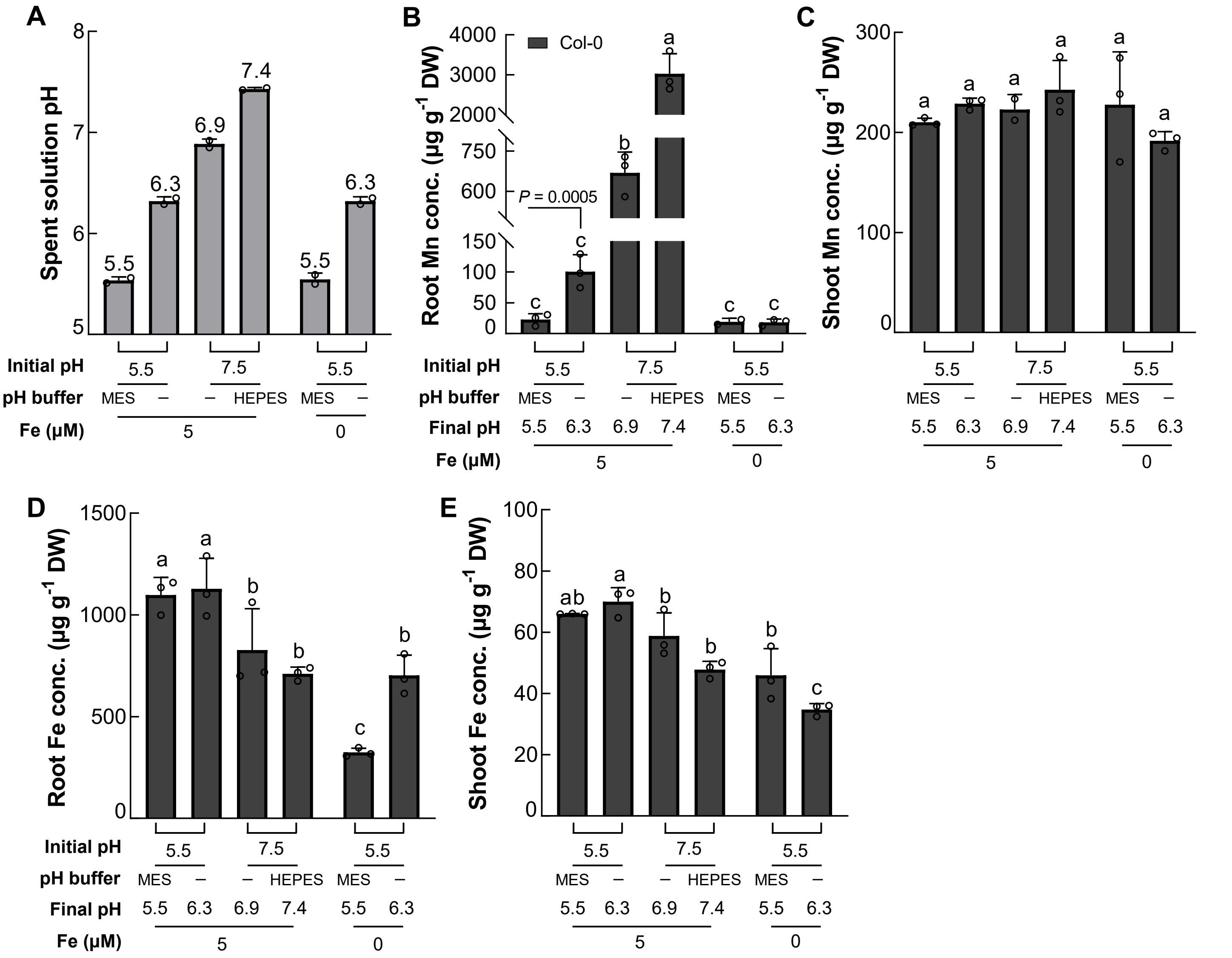

Fig. S12 Effect of pH buffering of hydroponic solutions on Mn and Fe homeostasis in *A. thaliana* exposed to low Fe and high pH. (**A**) pH measured in spent hydroponic solutions of five-week-old *A. thaliana* (Col-0) exposed to different initial pH and Fe levels for 6 d, with or without a pH buffer (at the time of solution exchange after 3 d of use). (**B–E**) Effects of initial pH, Fe supply and pH buffer on the concentrations of Mn (B, C) and Fe (D, E) in roots (B, D) and shoots (C, E) of *A. thaliana*. The pH of hydroponic solutions was either stabilized using buffers or left unbuffered, and solutions were exchanged every 3 d, with plant harvest after 6 d of treatment. Bars represent arithmetic mean ± SD of (n = 2 measurements after the first and second solution exchange (A), n = 3 replicate plants (B-E), in one culture vessel. Distinct letters denote statistically significant differences (p < 0.05) according to ANOVA (Duncan's test). Fe was supplied as a FeHBED complex. MES, 2-(N-morpholino)ethanesulfonic acid; HEPES, 4-(2-Hydroxyethyl)piperazine-1-ethanesulfonic acid.

Table S1 Linear regression models for the field data (Stein *et al.*, 2017).

Figure	Linear Regression Model	*df.residual*	*a*	*b*	*RSE*§	R^2	*F*-value	*P*-value
2D	Log_{10}(soil extr. Ca conc.) = *a* × Soil pH + *b*	1464	0.4116	4.121	0.2870	0.6457	2668.438	$< 2 \times 10^{-16}$
2E	Log_{10}(soil extr. Fe conc.) = *a* × Soil pH + *b*	1464	-0.4470	4.122	0.6405	0.3014	631.674	$< 2 \times 10^{-16}$
2F	Log_{10}(soil exch. Mn conc.) = *a* × Soil pH + *b*	1464	-0.5053	3.626	0.3873	0.6012	2207.407	$< 2 \times 10^{-16}$
S3A	Log_{10}(soil exch. Ca conc.) = *a* × Soil pH + *b*	1464	0.1184	2.167	0.3769	0.0804	127.943	$< 2 \times 10^{-16}$
S3B	Log_{10}(leaf Fe conc.) = a × Soil pH + b	1464	0.0134	2.182	0.2470	0.0026	3.816	0.0510
S3C	Leaf-to-soil extr. Fe conc. ratio = a × Soil pH + b	1464	0.4604	-1.940	0.6557	0.3040	639.464	$< 2 \times 10^{-16}$
S3D	Log_{10}(leaf Mn conc.) = *a* × Soil pH + *b*	1464	-0.1581	2.657	0.2301	0.2949	612.385	$< 2 \times 10^{-16}$
S3E	Leaf-to-soil exch. Mn conc. ratio = *a* × Soil pH + *b*	1464	0.3471	-0.969	0.4239	0.3728	870.102	$< 2 \times 10^{-16}$
S3F	Log_{10}(soil extr. S conc.) = *a* × Soil pH + *b*	1464	0.0670	0.766	0.2137	0.0802	127.688	$< 2 \times 10^{-16}$

§Residual Standard Error

Table S2 Linear regression models for experimental data from this study.

Figure	Linear Regression Model	Species	*df.residual*	*a*	*b*	*RSE*§	R^2	*F*-value	*P*-value
5D	Log_{10}(root Mn conc.) = *a* × Log_{10}(root hair no.) + *b*	*A. halleri*	20	1.872	-1.318	0.4963	0.3744	11.967	0.0025
		A. thaliana	13	2.471	-1.559	0.4594	0.6063	20.022	0.0006
S11A	Log_{10}(root Mo conc.) = *a* × Log_{10}(root hair no.) + *b*	*A. halleri*	20	0.075	0.293	0.3127	0.0024	0.049	0.8271
		A. thaliana	13	-0.970	2.372	0.1824	0.6009	19.571	0.0007
S11B	Log_{10}(root Mo conc.) = *a* × Log_{10}(root Mn conc.) + *b*	*A. halleri*	20	-0.267	1.088	0.2641	0.2963	9.264	0.0060
		A. thaliana	13	-0.329	1.600	0.1587	0.6980	30.044	0.0001

§Residual Standard Error

Table S3 Oligonucleotides used in the present study.

Oligo name	Gene	Sequence (5'-3')	Reference
qRT_UBQ10_Fw<br>qRT_UBQ10_Rv	*UBQ10*	GGCCTTGTATAATCCCTGATGAATAAG<br>AAAGAGATAACAGGAACGGAAACATAGT	Haydon et al., 2012
AtTIP41F<br>AtTIP41R	*TIP41-like*	GTGAAAACTGTTGGAGAGAAGCAA<br>TCAACTGGATACCCTTTCGCA	Han et al., 2013
bHLH39_At3g5698<br>bHLH39_At3g5698	*bHLH39*	CGTGACCGTCGCAGGAAAATTAAC<br>TCGCAGGAATGCTTAGCTTCTTCG	Quintana et al., 2021
qRT_bHLH101_Fw<br>qRT_bHLH101_Rv	*bHLH101*	CACCGCAAGGATCTTCTACAC<br>TCCCAAGAGCATACCATTGA	Quintana et al., 2021
qRT_FER1_Fw<br>qRT_FER1_Rv	*FER1*	TCCCCAGTTAGCTGATTTCG<br>CTTTGCCGATCATCCTTAGC	Grillet et al., 2018
qRT_IMA1_Fw<br>qRT_IMA1_Rv	*IRONMAN1*	TGATTGTAATTTAGGAGGAAACAAAA<br>TCAATCCACAAGTAAACATCTATGG	Grillet et al., 2018
qRT_ZIF1_Fw<br>qRT_ZIF1_Rv	*ZIF1*	ACAGTTGGACCAGCTGGTGC<br>GCTACTCCGACCACTACTATCAC	Quintana et al., 2021
qRT_BGLU42_Fw<br>qRT_BGLU42_Rv	*BGLU42*	ATGGCCTGGGAACTGAAGTC<br>ATTTGTCCAACCTCCGATTG	This study
qPCR_BTSL2_Fw<br>qPCR_BTSL2_Rv	*BTSL2*	CGGGGCAGAATCCATCTTAT<br>GTTGCAACAAGGAGCAAGAAG	Rodríguez-Celma et al., 2019
qRT_CYP71B5_Fw<br>qRT_CYP71B5_Rv	*CYP71B5*	GACGAGAGCCAATTGTTGGT<br>GAGGTTGGTCTCCGATTCAA	Quintana et al., 2021
qRT_IRT2_Fw<br>qRT_IRT2_Rv	*IRT2*	CGTAGCCATTGTTGCCATA<br>GGTTCCAAGGATGATTCCAG	Vert et al., 2009
qRT_MYB10_Fw<br>qRT_MYB10_Rv	*MYB10*	CGCTGGATTGATGAGATGCGGAA<br>TGAAGTTGCCTCGTTTGAGACCTG	Quintana et al., 2021

Literature cited

Han, B., Yang, Z., Samma, M. K., Wang, R., Shen, W. (2013) Systematic validation of candidate reference genes for qRT-PCR normalization under iron deficiency in Arabidopsis. Biometals **26**, 403-413.

Haydon MJ, Kawachi M, Wirtz M, Hillmer S, Hell R, Krämer U. (2012) Vacuolar nicotianamine has critical and distinct roles under iron deficiency and for zinc sequestration in Arabidopsis. Plant Cell **24**: 724–37.

Quintana, J., Bernal, M., Scholle, M., Holländer-Czytko, H., Nguyen, N. T., Piotrowski, M., Mendoza-Cozatl, D. G., Haydon, M. J., Krämer, U. (2022) Root-to-shoot iron partitioning in Arabidopsis requires IRON-REGULATED TRANSPORTER1 (IRT1) protein but not its iron (II) transport function. Plant J **109**, 992-1013.

Grillet L, Lan P, Li W, Mokkapati G, Schmidt W. (2018) IRON MAN is a ubiquitous family of peptides that control iron transport in plants. Nat Plants **4**: 953–963

Rodríguez-Celma J, Connorton JM, Kruse I, Green RT, Franceschetti M, Chen Y-T, Cui Y, Ling H-Q, Yeh K-C, Balk J. (2019) Arabidopsis BRUTUS-LIKE E3 ligases negatively regulate iron uptake by targeting transcription factor FIT for recycling. Proc Natl Acad Sci USA **116**: 17584–17591

Vert G, Barberon M, Zelazny E, Séguéla M, Briat J-F, Curie C. (2009) Arabidopsis IRT2 cooperates with the high-affinity iron uptake system to maintain iron homeostasis in root epidermal cells. Planta **229**: 1171–1179
